# Peroxisome proliferator-activated receptor alpha is an essential factor in enhanced macrophage immune function induced by angiotensin-converting enzyme

**DOI:** 10.1038/s41423-025-01257-y

**Published:** 2025-02-05

**Authors:** Suguru Saito, Duo-Yao Cao, Ellen A. Bernstein, Tomohiro Shibata, Anthony E. Jones, Amy Rios, Aoi O. Hoshi, Aleksandr B. Stotland, Erika E. Nishi, Jennifer E. Van Eyk, Ajit Divakaruni, Zakir Khan, Kenneth E. Bernstein

**Affiliations:** 1 Department of Biomedical Sciences, Cedars-Sinai Medical Center, Los Angeles, CA USA; 2 Department of Pathology and Laboratory Medicine, Cedars-Sinai Medical Center, Los Angeles, CA USA; 3 Department of Molecular and Medical Pharmacology, University of California, Los Angeles, Los Angeles, CA USA; 4 Graduate School of Comprehensive Human Science, University of Tsukuba, Tsukuba, Ibaraki Japan; 5 Department of Cardiology, Smidt Heart Institute, Cedars-Sinai Medical Center, Los Angeles, CA USA; 6 Department of Physiology, São Paulo School of Medicine, Universidade Federal de São Paulo, Sao Paulo, Brazil

**Keywords:** Angiotensin-converting enzyme, Macrophages, PPARα, Antitumor immunity, Bacterial clearance., Immunology, Infection, Tumour immunology

## Abstract

Increased expression of angiotensin-converting enzyme (ACE) by myeloid lineage cells strongly increases the immune activity of these cells, as observed in ACE10/10 mice, which exhibit a marked increase in antitumor and antibactericidal immunity. We report that peroxisome proliferator-activated receptor alpha (PPARα), a transcription factor that regulates genes critical for lipid metabolism, is a key molecule in the enhanced macrophage function induced by ACE. Here, we used a Cre–LoxP approach with LysM-Cre to create a modified ACE10/10 mouse line in which macrophages continue to generate abundant ACE but in which monocyte and macrophage PPARα expression is selectively suppressed. These mice, termed A10-PPARα-Cre, have significantly increased growth of B16-F10 tumors compared with ACE10/10 mice with Cre expression. PPARα depletion impaired cytokine production and antigen-presenting activity in ACE-expressing macrophages, resulting in reduced tumor antigen-specific CD8^+^ T-cell generation. Additionally, the elevated bactericidal resistance typical of ACE10/10 mice was significantly reduced in A10-PPARα-Cre mice, such that these mice resembled WT mice in their resistance to methicillin-resistant *Staphylococcus aureus* (MRSA) infection. THP-1 cells expressing increased ACE (termed THP-1-ACE) constitute a human macrophage model with increased PPARα that shows enhanced cytotoxicity against tumor cells and better phagocytosis and killing of MRSA. RNA silencing of PPARα in THP-1-ACE cells reduced both tumor cell death and bacterial phagocytosis and clearance. In contrast, the in vivo administration of pemafibrate, a specific agonist of PPARα, to WT and A10-PPARα-Cre mice reduced B16-F10 tumor growth by 24.5% and 25.8%, respectively, but pemafibrate reduced tumors by 57.8% in ACE10/10 mice. With pemafibrate, the number of antitumor CD8^+^ T cells was significantly lower in A10-PPARα-Cre mice than in ACE10/10 mice. We conclude that PPARα is important in the immune system of myeloid cells, including wild-type cells, and that its increased expression by ACE-expressing macrophages in ACE10/10 mice is indispensable for ACE-dependent functional upregulation of macrophages in both mice and human cells.

## Introduction

Recent studies have indicated that fatty acid metabolism is important in the functional regulation of macrophages [1–3]. Our group has studied the immune response of a genetically modified mouse model called ACE10/10, in which monocytes and macrophages express increased angiotensin-converting enzyme (ACE). In these mice, both macrophage function and the overall immune response to bacterial infection, cancer, atherosclerosis, and Alzheimer’s disease are much more vigorous and effective than they are in control animals [4–11].

While studying the mechanism by which ACE affects myeloid function, increased ACE expression clearly induced a marked change in the metabolism of these cells characterized by increased lipid utilization via mitochondrial oxidative metabolism and increased content of adenosine triphosphate (ATP) in the cells [12]. Importantly, increased macrophage ACE expression triggers the upregulation of peroxisome proliferator-activated receptor alpha (PPARα), a transcription factor central to lipid metabolism in cells [9, 10]. With ligand stimulation, PPARα forms a heterodimer with the retinoid X receptor (RXR), which is then recruited into the nucleus and binds to PPAR response elements (PPREs) to trigger the expression of dozens of genes responsible for cytoplasmic lipid handling and mitochondrial metabolism [13–15]. These metabolic pathways utilize various classes of lipids, such as cholesterol, triglycerides, phospholipids, bile acids, and fatty acids [16]. Hepatocytes express relatively high levels of PPARα and serve as a model system for investigating the role of PPARα in lipid metabolism [15, 16]. This transcription factor is also expressed in myeloid cells, such as monocytes and macrophages, although the importance of this protein in cell metabolism and immune function has received much less attention [17]. The limited information available indicates that PPARα affects macrophage functions, such as intracellular lipid clearance and reverse lipid transport [16, 17]. One reason for the difficulty in elucidating the functional importance of PPARα in immune cells, including macrophages, is the relatively lower expression levels in these cells than in hepatocytes [18]. While the analysis of ACE10/10 mice revealed that increased macrophage ACE expression results in increased cell PPARα, a marked change in lipid metabolism, and increased macrophage function [9, 10], the key question of whether increased PPARα is directly responsible for the increased immune response of these macrophages remains unanswered.

To investigate this, we used a Cre–LoxP approach to create a modified ACE10/10 mouse line in which macrophages continue to generate abundant ACE but in which monocyte and macrophage PPARα expression is selectively suppressed. Compared with parent ACE10/10 cells, this genetic modification resulted in marked downregulation of macrophage fatty acid metabolism, ATP production, and the immune response of macrophages. Our data suggest that PPARα is the central functional regulator of the enhanced immune capacity exhibited by ACE10/10 mice. Furthermore, these data highlight for the first time that increased PPARα activity is a potent target for enhancing macrophage function and indeed increasing the function of the entire immune system.

## Results

### Selective depletion of PPARα in ACE10/10 mice

**Fig. 1:**
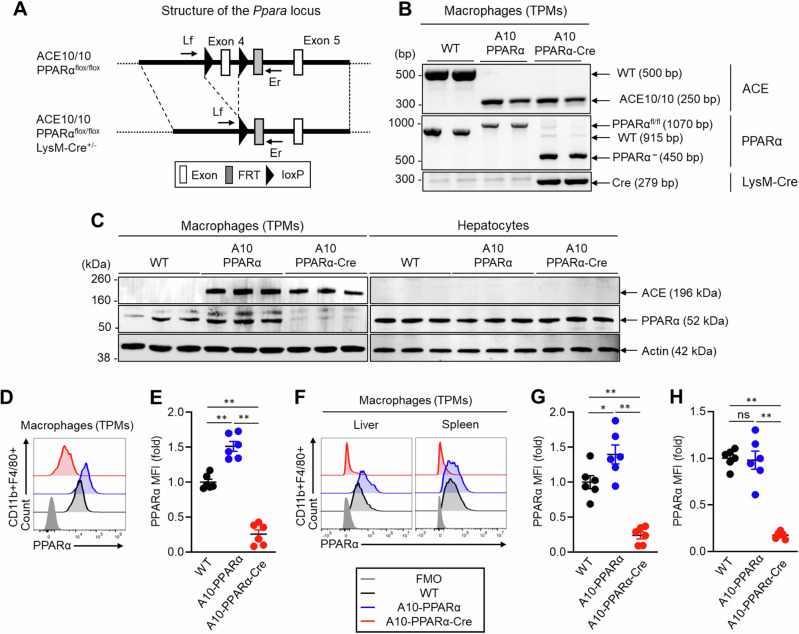
Genetic design of macrophage-specific PPARα deletion in ACE10/10 mice. **A** A10-PPRAα mice are ACE10/10 mice in which exon 4 of both PPARα genes is floxed (see “Methods” section). This construct was crossed with LysM-Cre mice to obtain A10-PPRAα-Cre mice (conditional KO), in which cre recombinase, expressed by macrophages and monocytes, excises PPARα exon 4, functionally eliminating this protein. To measure the depletion of exon 4, PCR was performed by using the primers Lf and Er (Supplementary Table 1). **B** Representative gel images of the WT, A10-PPARα, and A10-PPRAα-Cre mice generated by genotyping PCR. **C** PPARα expression in thioglycolate-elicited peritoneal macrophages (TPMs) and hepatocytes was measured by Western blotting. **D**, **E** Representative histogram (**D**) and MFI values (fold change) (**E**) of PPARα expression in TPMs measured by flow cytometry. **F**–**H** Representative histogram (**F**) and PPARα MFI values (fold change) in the spleen (**G**) and liver (**H**) resident macrophages measured by flow cytometry. The cumulative data are shown as the mean ± SEM values of six samples from two independent experiments. All MFI values are represented as fold changes (the average value of WT was used for a value equal to 1). One-way ANOVA was used to analyze the significance of the data. *p < 0.05 and **p < 0.001; ns not significant

The generation and characterization of ACE10/10 and ACE knockout (KO) mice were described in previous reports [4–12]. An earlier study revealed that PPARα expression was elevated in thioglycolate-elicited peritoneal macrophages (TPMs) from ACE10/10 mice and reduced in equivalent cells from ACE-KO mice compared with wild-type (WT) macrophages. Indeed, there was a direct correlation between PPARα expression and the expression of antigen presentation-related molecules (CD80, CD86, H-2K^b^, and I-A^b^), phagocytosis, antigen-dependent stimulation of T cells, and cytokine production (Supplementary Fig. 1A–K). To further investigate the role of PPARα in ACE10/10 macrophages, we studied 4 groups of mice: wild type (WT), ACE10/10 in which exon 4 of the PPARα gene is homozygously floxed (termed A10-PPARα), A10-PPARα mice heterozygous for the myeloid-specific cre expressor LysM-Cre (termed A10-PPARα-Cre mice) (Fig. 1A, B), and ACE10/10 mice heterozygous for the LysM-Cre gene but having a nonfloxed wild-type PPARα gene (termed ACE10/10-Cre) to study whether the loss of a single allele of the Lyz2 gene, as well as the presence of Cre recombinase, affects macrophage immune function (Supplementary Fig. 2).

Initial characterization of PPARα protein expression was performed via western blotting (WB) of TPMs and hepatocytes from WT, A10-PPARα, and A10-PPARα-Cre mice. PPARα expression was increased in TPMs from A10-PPARα mice compared with those from WT mice (approximately 36%), whereas its expression was downregulated in the TPMs of A10-PPARα-Cre mice (approximately 87%, Fig. 1C, left). In contrast, this depletion strategy did not affect PPRAα protein expression by hepatocytes (Fig. 1C, right). We also investigated PPARα expression in bone marrow (BM)-derived neutrophils, which may be affected by the LysM-Cre-mediated gene depletion strategy [19], but in our strains of mice, neutrophil PPARα protein expression was equivalent among the three genetic backgrounds (Supplementary Fig. 3). We also measured PPARα expression in TPMs via flow cytometry. This analysis revealed 83% and 75% reductions in the mean fluorescence intensity (MFI) of PPARα in TPMs from A10-PPARα-Cre mice compared with either A10-PPARα cells or WT cells (Fig. 1D, E). PPARα expression was also investigated in tissue-resident macrophages via flow cytometry. Compared with that in WT cells, the MFI of PPARα was decreased by more than 80% in the splenic and liver macrophages of A10-PPARα-Cre mice (Fig. 1F–H). Interestingly, PPARα expression was increased by 40% in the liver macrophages of A10-PPARα mice compared with those of WT mice; however, the number of splenic macrophages did not change (Fig. 1F–H).

Global immune screening was performed to compare the basal immune environment among the three groups of mice. We analyzed the percentages of lymphocytes (T cells and B cells) and myeloid cells (macrophages, monocytes, dendritic cells, and neutrophils) in the spleen, liver, lymph nodes (LNs), bone marrow (BM), and peripheral blood (PB). PPARα depletion did not affect the frequency of these cells in A10-PPARα-Cre mice compared with A10-PPARα and WT mice (Supplementary Fig. 4A–E). However, the frequencies of IFN-γ^+^CD4^+^ T cells (Th1), IL-17A^+^CD4^+^ T cells (Th17), and IFN-γ^+^CD8^+^ T cells (Tc1) were mildly greater in the spleens of A10-PPARα mice than in those of WT and A10-PPARα-Cre mice (Supplementary Fig. 4F).

### Gene expression profile of PPARα-depleted ACE-OE macrophages

To characterize the change in gene expression induced by the reduction in macrophage PPARα expression, TPMs from WT, A10-PPARα, and A10-PPARα-Cre mice were examined via bulk RNA sequencing. This analysis revealed that the reduction in PPARα expression in ACE-overexpressing macrophages results in a marked change in the gene expression pattern, with a PCA plot showing 3 distinct gene clusters (Supplementary Fig. 5). Specifically, a comparison via Bland‒Altman plots (MA plots) of the number of genes in which there were differences in RNA expression among the three groups studied revealed that the comparison of A10-PPARα vs. WT cells revealed 2276 differences in gene expression (up: 1946 and down: 330), A10-PPARα vs. A10-PPARα-Cre revealed 1811 differences (up: 1409 and down: 402), while A10-PPARα-Cre vs. WT identified only 1499 differences (up: 1078 and down: 421; differences were defined as fold change (FC) ≥ 2 or FC ≤ 0.5 in gene expression, Supplementary Fig. 6A‒C). This finding suggested that A10-PPARα-Cre macrophages more closely resembled WT cells than A10-PPARα cells did.

**Fig. 2:**
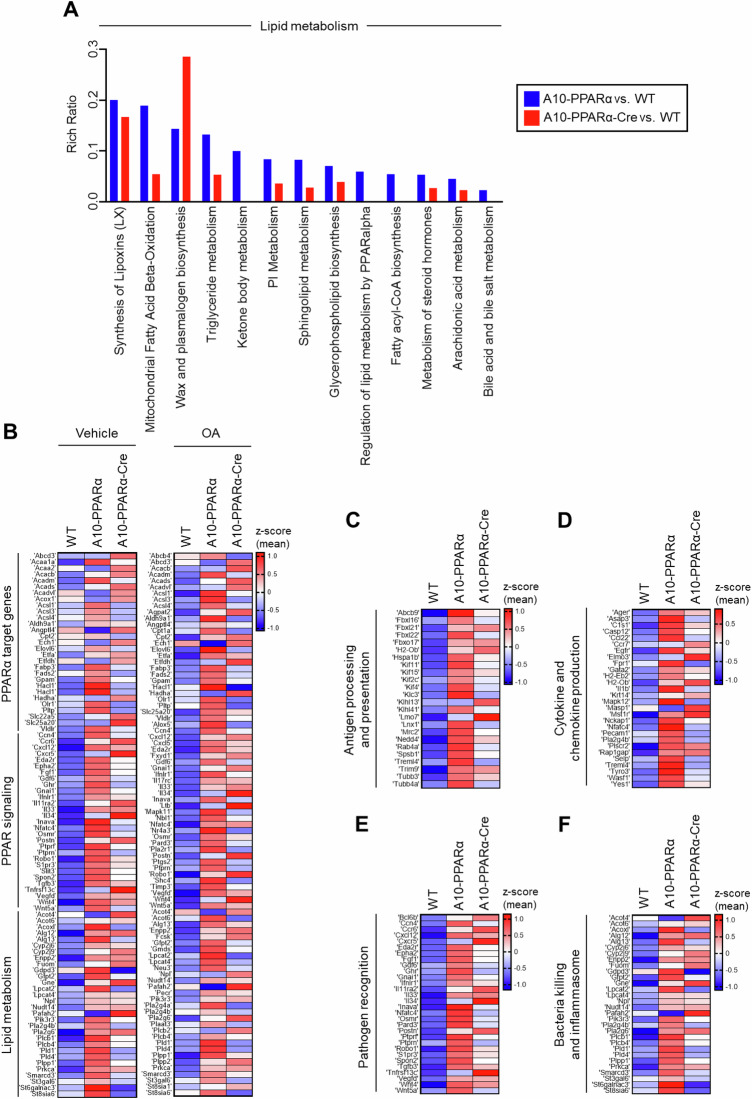
PPARα depletion alters the expression of lipid metabolism- and immune response-associated genes in macrophages. Total RNA was isolated from thioglycolate-elicited peritoneal macrophages of WT, A10-PPARα, or A10-PPARα-Cre mice (*n* = 3 in each) and subjected to bulk RNA sequencing. As shown in (**B**), some of these macrophages were incubated with vehicle (ethanol) or OA (200 µM) at 37 °C for 36 h before total RNA isolation. The TPM values obtained from RNA sequencing were used for the analyses via conversion to log2(TPM + 1) values. **A** Lipid metabolism-associated pathways were determined by Ingenuity Pathway Analysis (IPA) using genes identified as having significant expression changes (*p* < 0.05) in either of the two independent comparisons: A10-PPARα-Cre vs. WT and A10-PPARα-Cre vs. WT. The Rich Raito of each pathway was also calculated via IPA. The figure shows identified pathways in which the Rich ratio for A10-PPARα-Cre vs. WT is either greater than 1.2-fold or less than 0.8-fold greater than that of A10-PPARα-Cre vs. WT. Additionally, pathways with a Rich ratio of 0 for A10-PPARα-Cre vs. WT are also shown. Individual values for the pathways shown here and all pathways analyzed are shown in Supplementary Table 6. **B**–**F** Expression profile of genes associated with lipid metabolism and the immune system. The gene sets belonging to the categories shown were extracted via the KEGG pathway database. The mean log2(TPM + 1) values of the WT were compared to those of A10-PPARα or A10-PPARα-Cre independently, and the 20 genes with the greatest increase in expression from the WT for each comparison were extracted to generate heatmaps. z scores were calculated from the log2(TPM + 1) values and used to visualize gene expression differences in the heatmaps. The gene expression differences of these pathways, including PPARα target genes, PPAR signaling, lipid metabolism (with vehicle or OA treatment) (**B**), antigen processing and presentation (**C**), cytokine and chemokine production (**D**), pathogen recognition (**E**), and bacterial killing and inflammasome formation (**F**), are presented as heatmaps

When only genes whose gene expression differed from the WT values were compared (*p* < 0.05), Ingenuity Pathway Analysis (IPA) identified two physiologic areas—genes associated with lipid metabolism and the immune system—as enriched for such differences (Fig. 2A, Supplementary Fig. 7, and Supplementary Table 6). These figures show the output of IPA in classifying genes according to functional pathways. For each pathway, the Rich ratio, which is the number of genes within the pathway exhibiting a significant difference in gene expression compared with the WT value, divided by the total number of genes in that pathway, was determined via software. A greater Rich ratio represents a more pronounced difference between gene sets. A comparison of the Rich ratios for the two comparisons A10-PPARα vs. WT and ACE10-PPARα-Cre vs. WT again suggested that WT macrophages more closely resembled A10-PPARα-Cre cells than A10-PPARα cells. For example, the Rich ratio for the pathway ‘mitochondrial fatty acid β-oxidation’ was 0.189 for the A10-PPARα vs. WT comparison, a value 3.5-fold greater than the Rich ratio of 0.054 for A10-PPARα-Cre vs. WT (Fig. 2A, Supplementary Table 6). In terms of lipid metabolism, 12 pathways presented a rich ratio that was at least 1.2-fold greater in the A10-PPARα vs. WT comparison than in the A10-PPARα-Cre vs. WT comparison. Conversely, only 1 pathway had a 1.2-fold greater Rich ratio in the A10-PPARα-Cre group than in the WT group (Fig. 2A). Similarly, for immune system pathways, 23 pathways presented Rich ratios that were at least 1.2-fold greater in the A10-PPARα than in the WT comparison, whereas 20 pathways presented the opposite trend, with Rich ratios that were 0.8-fold greater or lower in the A10-PPARα than in the WT comparison than in the A10-PPARα-Cre vs. WT comparison (Supplementary Fig. 7).

Given the known role of PPARα in regulating lipid metabolism genes, we examined whether the gene expression patterns changed between WT, A10-PPARα, and A10-PPARα-Cre TPMs when the cells were exposed to oleic acid (OA), a monounsaturated omega-9 fatty acid. The heatmaps show the relative expression levels of the 20 genes whose expression increased the most from that of the WT for the KEGG categories ‘PPARα target genes’, ‘PPAR signaling’, and ‘Lipid metabolism’ (Fig. 2B). These data show that under basal conditions and after OA, the upregulation of A10-PPARα z scores in the A10-PPARα vs. WT gene set was more pronounced than the ACE10-PPARα-Cre z scores of the same genes. To quantitate this, we again compared the TPM values for these genes. Compared with ACE10-PPARα-Cre cells, 31 genes from A10-PPARα cells presented a 1.2-fold or greater increase in TPM; only 6 genes presented a 1.2-fold increase in ACE10-PPARα-Cre compared with ACE10-PPARα. Analysis of the OA gene set revealed very similar results: the expression of 30 genes increased by 1.2-fold or more when ACE10-PPARα was compared with ACE10-PPARα-Cre, whereas the expression of only 8 genes showed the opposite result.

Figure 2C–F show the expression levels of genes associated with both innate and adaptive immunity in TPMs, such as ‘antigen processing and presentation’, ‘cytokine and chemokine production’, and ‘pathogen recognition and bactericidal effects’. For each of these categories, the conclusions are similar to those for the categories in Fig. 2B: A10-PPARα macrophages expressed more transcripts than either WT or A10-PPARα-Cre macrophages did. In fact, all the heatmap presentations and pathway comparisons indicate that the inability of A10-PPARα-Cre macrophages to express PPARα makes these cells resemble WT macrophages far more than A10-PPARα cells do.

### Metabolic features of PPARα-depleted ACE-OE macrophages

**Fig. 3:**
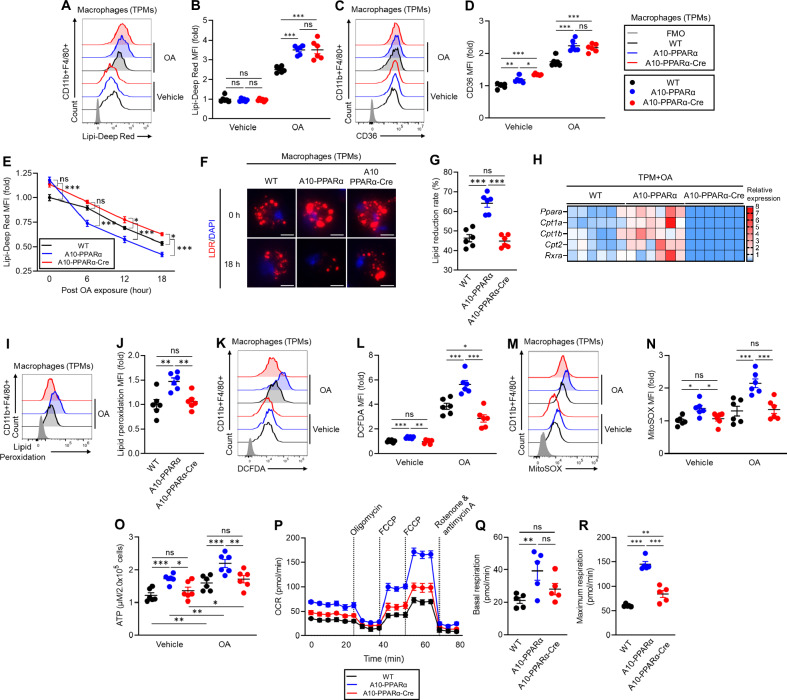
PPARα depletion alters lipid metabolism in ACE-overexpressing macrophages. **A**–**G** TPMs were incubated with vehicle (ethanol) or 200 μM OA for 16 h. **A**, **B** In vitro lipid uptake assay. The intracellular lipid content was determined via Lipi-Deep Red (LDR) staining followed by flow cytometry analysis. Representative histograms (**A**) and MFI values (fold change) (**B**) of LDR signals in TPMs. **C**, **D** CD36 expression. Representative histograms (**C**) and MFI values (fold change) (**D**) of CD36 expression in TPMs measured by flow cytometry. **E**–**G** Lipid consumption. TPMs were treated with 200 μM OA at 37 °C for 16 h and then washed and cultured in media (without OA) for 6, 12, or 18 h. Intracellular lipids were stained with LDR and quantified via flow cytometry at each time point. **E** Time-dependent lipid reduction. **F** Fluorescence microscopy images of intracellular lipids in TPM immediately following OA loading (0 h) or after 18 h without OA. **G** Lipid reduction rate at 18 h post-OA exposure. **H** Gene expression profile of OA-treated TMPs. The TPM samples were cultured with 200 μM OA for 16 h, after which the isolated total RNA was subjected to real-time PCR. Gene expression was quantified via the ∆Ct method. **I**, **J** Lipid peroxidation. TPMs were incubated with 200 μM OA for 16 h, and lipid peroxidation was subsequently measured by staining with a peroxidation probe and flow cytometry. Representative histogram (**I**) and MFI values (fold change) (**J**) of lipid peroxidation. **K**, **L** ROS production. TPMs were incubated with ethanol or 200 μM OA for 16 h. Cytosolic ROS and mitochondrial ROS (mtROS) were measured by flow cytometry with H_2_DFCDA and MitoSOX^TM^, respectively. Representative histograms (**K**) and MFI values (fold change) (**L**) of cytosolic ROS levels in TPMs. Representative histograms (**M**) and MFI values (fold change) (**N**) of mtROS levels. **O** Measurement of the intracellular ATP concentration. TPMs were incubated with ethanol or OA as described above, and then, intracellular ATP was quantified via the luminescence-based assay CellTiter-Glo 2.0. The ATP concentrations were calculated via the standard curve method. **P**–**R** Real-time metabolic analysis of TPM by Seahorse. **P** Transition of the oxygen consumption rate (OCR) in the TPMs during analysis. **Q**, **R** Basal respiration and maximal respiration of TPMs. The cumulative data are shown as the means ± SEMs of five to six samples from two independent experiments. All MFI values are represented as fold changes (the average value of WT was used for base = 1). One-way ANOVA was used to analyze the significance of the data. *p < 0.05, **p < 0.01 and ***p < 0.01. ns indicates not significant

To examine whether PPARα depletion affects the metabolic characteristics of macrophages, we performed in vitro experiments using TPMs prepared from WT, A10-PPARα, and A10-PPARα-Cre mice. A lipid uptake assay was performed by culturing TPMs with or without OA, after which intracellular lipid droplets (LDs) were stained with the lipid-specific dye Lipi-Deep Red (LDR). The LDR signals were measured via flow cytometric analysis (Fig. 3A, B). Without OA treatment, the basal lipid content was similar among the three groups of cells. However, OA exposure significantly increased the LDR signal in both the A10-PPARα and A10-PPARα-Cre TPMs compared with that in WT cells; thus, PPARα did not affect OA uptake. In the same experiment, we also measured the expression of the scavenger receptor CD36, which can capture extracellular fatty acids (Fig. 3C, D) [20]. CD36 expression was significantly greater in A10-PPARα TPMs than in WT cells, even under basal conditions. CD36 expression in A10-PPARα-Cre TPMs was equivalent to that in A10-PPARα TPMs, implying that the expression of this receptor is also not affected by the expression level of PPARα in ACE-overexpressing macrophages. OA treatment elevated CD36 expression in all three groups of TPMs, but the difference between WT and ACE-overexpressing macrophages (regardless of PPARα expression) was pronounced.

We next investigated whether depletion of PPARα affects lipid consumption in macrophages. TPMs from the three groups of mice were cultured with OA for 16 h, after which intracellular lipids were measured via LDR staining and flow cytometry at 6, 12, and 18 h postlipid exposure. By comparing the slopes of these curves and the final amount of intracellular lipids, we were able to estimate the ability of each population of macrophages to metabolize intracellular lipids (Fig. 3E). As we previously reported, ACE10/10 macrophages (equivalent here to A10-PPARα) have an enhanced ability to clear intracellular lipids compared with WT cells [9, 10]. In contrast, the reduction in PPARα in A10-PPARα-Cre TPM resulted in a substantial reduction in the rate at which macrophages can clear intracellular lipids. Representative fluorescence microscopy images are consistent with the flow cytometry data, which revealed significantly fewer intracellular lipid droplets in A10-PPARα cells after 18 h than in WT or A10-PPARα-Cre macrophages (Fig. 3F). After 18 h, the mean lipid reduction in A10-PPARα TPMs was approximately 65%, whereas both WT and A10-PPARα-Cre TPMs reduced lipid levels by less than 50% (Fig. 3G).

Given these findings, we analyzed the expression of fatty acid metabolism-associated genes in TPMs treated with OA. Specifically, genes regulated by PPARα, such as carnitine palmitoyl transferase 1A (*Cpt1a*), *Cpt1b*, *Cpt2*, and *Rxra*, were analyzed via real-time qPCR and consistently presented increased mRNA levels in A10-PPARα TPMs compared with WT cells. In contrast, A10-PPARα-Cre TPMs not only had fewer mRNAs than A10-PPARα cells but also expressed lower levels of these genes than WT TPMs did (Fig. 3H).

We previously reported that ACE10/10 macrophages produce more reactive oxygen species (ROS) than WT cells do under basal conditions and following lipid exposure, suggesting that ACE induces more effective lipid oxidative metabolism [9, 10, 12]. To examine the role of PPARα, we tested TPMs from the three groups of mice for lipid peroxidation, ROS, and ATP levels. These findings revealed that the level of lipid peroxidation was significantly greater in A10-PPARα TPMs than in WT cells upon OA exposure. A reduction in PPARα in A10-PPARα-Cre cells decreased lipid peroxidation to levels equivalent to those in WT TPMs (Fig. 3I, J). Compared with the other two groups, A10-PPARα TPMs also presented increased intracellular ROS levels, even under basal conditions. OA treatment increased ROS production in all three groups, with A10-PPARα TPMs maintaining the highest production level (Fig. 3K, L). Mitochondrial ROS (mtROS) production followed the same trend, with A10-PPARα TPMs producing significantly higher levels under both basal and OA-exposed conditions than the other two groups of TPMs did (Fig. 3M, N). The intracellular ATP content was also significantly greater in A10-PPARα TPMs than in the other two groups, even under basal conditions. The ATP concentration significantly increased in all three groups of TPMs with OA treatment, but A10-PPARα TPMs still produced the highest ATP levels (Fig. 3O).

Finally, we analyzed mitochondrial activity in the three groups of TPMs by measuring the oxygen consumption rate (OCR) via a Seahorse analyzer. As predicted on the basis of the levels of intracellular ATP, both basal and maximum mitochondrial respiration were significantly greater in A10-PPARα TPMs than in WT cells. In contrast, the loss of PPARα expression in A10-PPARα-Cre TPMs was associated with a basal respiration rate similar to that of WT cells, and the maximum respiration rate significantly decreased compared with that of A10-PPARα, although it was still somewhat elevated compared with that of WT cells (Fig. 3P–R).

In our analysis, we measured the intracellular levels of several metabolites in TPMs. Whole metabolome analysis via a partial least squares-discriminant analysis (PLS-DA) plot revealed that each of the three groups (A10-PPARα, A10-PPARα-Cre, and WT TPMs) had a distinct metabolome, with that of the A10-PPARα-Cre cells shifting toward the WT cell metabolome (Supplementary Fig. 8A). Additionally, some of the metabolites associated with mitochondrial oxidative metabolism were increased in A10-PPARα TPMs compared with both A10-PPARα-Cre and WT cells (Supplementary Fig. 8B).

We also measured ATP and ROS levels as well as lipid uptake activity in TPMs from ACE10/10-Cre mice that express LysM-Cre but without the floxed PPARα construct (i.e., with the WT PPARα gene). All the results were comparable between ACE10/10 and ACE10/10-Cre TPMs (Supplementary Fig. 2A–C), indicating that the expression of Cre recombinase and the lack of a single allele of the *Lyz2* gene were not the causes of the downregulation of metabolic activity in A10-PPARα-Cre cells.

### Reduced antitumor immunity in A10-PPARα-Cre mice

**Fig. 4:**
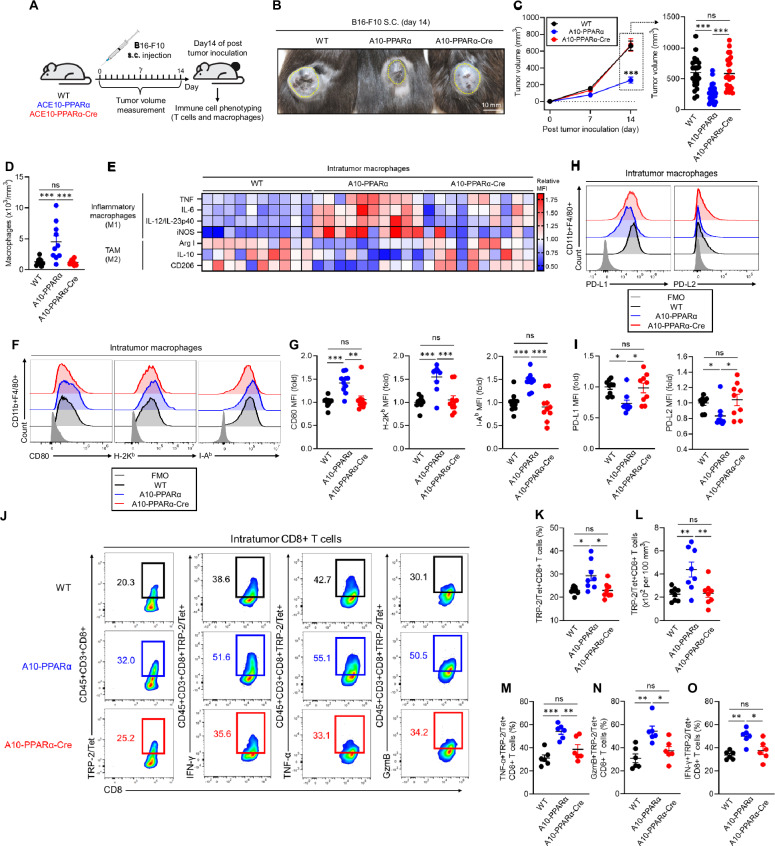
PPARα depletion impairs the antitumor activity of A10-PPARα-Cre mice. **A** Experimental design of the murine B16-F10 tumor model. The mice received a subcutaneous (s.c.) injection of B16-F10 cells (100 μL of 1.0 × 10^7^/mL in PBS). The tumor volumes were measured, and the immunological activities of intratumor (IT) macrophages and CD8^+^ T cells were analyzed via flow cytometry on day 14 posttumor inoculation. **B** Representative pictures of tumors. **C** Tumor volumes. **D** Number of tumor-infiltrating macrophages. **E** Functional marker expression of IT macrophages. M1 markers (TNF-α, IL-6, IL-12/IL-23p40, and iNOS) and M2 markers (arginase 1 (Arg 1), IL-10, and CD206) were measured via flow cytometry, and MFI values were used to generate a heatmap. **F**, **G** Representative histograms (**H**) and MFI values (fold change) (**I**) of CD80, H-2K^b^, and I-A^b^ expression in IT macrophages. **H**, **I** Representative histograms (**F**) and MFI values (fold change) (**G**) of PD-L1 and PD-L2 expression by IT macrophages. **J**–**O** Functional characterization of IT CD8^+^ T cells. **J** Representative plots of TRP-2/tetramer (Tet)^+^CD8^+^ T cells and IFN-γ^+^, TNF-α^+^, or GzmB^+^ populations among TRP-2/Tet^+^CD8^+^ T cells. **K**, **L** Percentages (**K**) and cell numbers (per 100 mm^3^ of tumor) (**L**) of TRP-2/Tet^+^CD8^+^ T cells. **M**–**O** Percentages of TNF-α^+^ (**M**), IFN-γ^+^ (**N**), or GzmB^+^ (**O**) TRP-2/Tet^+^CD8^+^ T cells. The cumulative data are shown as the means ± SEMs of six to ten samples from two or three independent experiments. All MFI values are represented as fold changes (the average value of WT was used for base = 1). One-way ANOVA was used to analyze the data for significant differences. *p < 0.05, **p < 0.01, and ***p < 0.001; ns not significant

To investigate the functional role of PPARα in the immune response, we challenged WT, A10-PPARα, and A10-PPARα-Cre mice with subcutaneous injections of murine melanoma B16-F10 cells. In this model, tumor growth was tracked during the experimental period, and all surviving mice were sacrificed on day 14 after tumor inoculation for analysis. The excised tumors were analyzed by immune phenotyping of macrophages and T cells in the tumor microenvironment (TME) via flow cytometry (Fig. 4A). We previously reported that ACE10/10 mice have a substantially better antitumor response than WT mice do and display smaller tumors at sacrifice [6]. A similar result was observed in A10-PPARα mice; the tumors were significantly smaller than those in WT mice (Fig. 4B, C). In contrast, the tumor volume in A10-PPARα-Cre mice was significantly greater than that in A10-PPARα mice and was similar to that in WT mice.

During the B16‒F10 challenge in ACE10/10 mice, many of the characteristics associated with proinflammatory M1 macrophages are increased [6]. This pattern was reproduced in A10-PPARα mice. Specifically, compared with WT animals, A10-PPARα mice have more macrophages within the tumor (per mm³ of tumor) (Fig. 4D), and these intratumor (IT) macrophages express increased levels of cytokines such as tumor necrosis factor α (TNF-α), interleukin-6 (IL-6), IL-12/IL-23p40, and inducible nitric oxide synthase (iNOS) (Fig. 4E). IT macrophages derived from A10-PPARα-treated cells expressed lower levels of arginase I (Arg I), IL-10, and CD206 than WT cells did (Fig. 4E). Remarkably, for all these measures of increased inflammatory activity, A10-PPARα-Cre macrophages have a phenotype equivalent to that of WT cells.

As indicated, the A10-PPARα mice had more inflammatory IT macrophages than did the WT mice. These cells expressed increased levels of CD80, H-2K^b^ (MHC class I), and I-A^b^ (MHC class II) molecules, indicating the potential for better antigen presentation to T cells (Fig. 4F, G). Additionally, A10-PPARα macrophages expressed lower levels of the immunosuppressive molecules programmed death-ligand 1 (PD-L1) and PD-L2 than did WT and A10-PPARα-Cre macrophages in the TME (Fig. 4H, I) [21, 22]. This effect appears to result in a better CD8^+^ T-cell tumor response, as indicated by a greater frequency and number of IT CD8^+^ T cells specific for the melanoma tumor antigen tyrosinase-related protein-2 (TRP-2) (TRP-2/Tet^+^CD8^+^ T cells) (Fig. 4J–L) and higher expression of TNF-α, IFN-γ, and granzyme B (GzmB) in TRP-2/Tet^+^CD8^+^ T cells (Fig. 4J, M–O). The frequency of IFN-γ^+^ or TMF-α^+^CD4^+^ T cells was also significantly greater in the TME of A10-PPARα mice than in that of WT and A10-PPARα-Cre mice (Supplementary Fig. 10A, B). However, the A10-PPARα-Cre mice were essentially equivalent to the WT mice for all the measured CD8^+^ T-cell responses.

**Fig. 5:**
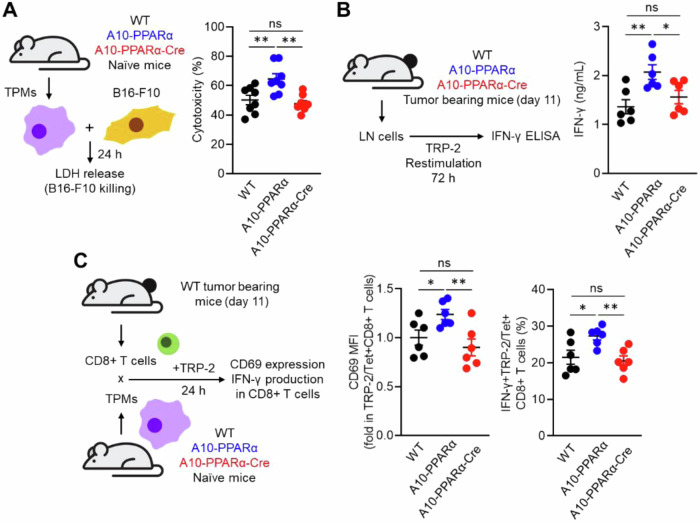
PPARα depletion attenuates tumor killing and the antigen-presenting ability of ACE-overexpressing macrophages. **A** In vitro tumor-killing assay. B16-F10 cells and TPMs prepared from WT, A10-PPARα or A10-PPARα-Cre naïve mice were mixed at a 1:1 ratio and incubated at 37 °C for 24 h. LDH levels in the cultures were measured via absorbance, and the values were used to calculate the tumor-killing rates. **B** In vitro antigen restimulation assay. Inguinal lymph node (iLN) cells were isolated from tumor-bearing mice 11 days after tumor inoculation and were restimulated with TRP-2 peptide (100 μg/mL) at 37 °C for 72 h. IFN-γ concentrations in the culture medium were measured via ELISA. **C** In vitro antigen presentation assay. CD8^+^ T cells were isolated from the iLNs of tumor-bearing WT mice 11 days after tumor inoculation. TPMs were also prepared from naive WT, A10^-^PPARα, or A10-PPARα-Cre mice and cocultured with CD8^+^ T cells in the presence of the TRP-2 peptide (100 μg/mL) at 37 °C for 24 h. The expression (MFI) of CD69 and the percentages of IFN-γ-producing cells in the CD8^+^ T-cell population were measured via flow cytometry. The cumulative data are shown as the mean ± SEM of six samples from two independent experiments. All MFI values are represented as fold changes (the average value of WT was used as 1). One-way ANOVA was used to analyze the data for significant differences. *p < 0.05 and **p < 0.01; ns not significant

We also performed in vitro functional assays of macrophages. The first is the ability of macrophages to kill B16-F10 tumor cells. For this experiment, TPMs from WT, A10-PPARα, or A10-PPARα-Cre mice were mixed with B16-F10 tumor cells, and the direct tumor-killing activity of the TPMs was assessed by measuring lactate dehydrogenase (LDH) release [23]. The tumor-killing effect of A10-PPARα TPMs was greater than that of equivalent cells from WT mice, whereas PPARα reduction in A10-PPARα-Cre TPMs was similar to that in WT cells (Fig. 5A). We also performed an in vitro T-cell restimulation assay to estimate the abundance of tumor antigen-specific T cells. B16-F10 tumor cells were inoculated into the 3 groups of mice, and inguinal lymph node (iLN) cells were isolated on day 11 posttumor inoculation. The iLN cells were restimulated with the TRP-2 peptide, and the tumor antigen-specific T-cell response was assessed via INFγ production. The iLN cells from the A10-PPARα mice produced more IFN-γ in response to TRP-2 stimulation than the WT cells did, whereas the response of the A10-PPARα-Cre iLN cells was equivalent to that of the WT cells (Fig. 5B).

Finally, we performed an antigen presentation assay using TPMs and tumor-sensitized CD8^+^ T cells. CD8^+^ T cells were isolated from the iLNs of tumor-bearing WT mice after 11 days of B16‒F10 inoculation. These CD8^+^ T cells were cocultured with TPMs from WT, A10-PPARα, or A10-PPARα-Cre mice in the presence of the TRP-2 peptide. After incubation for 24 h, we measured the expression of CD69 and IFN-γ in TRP-2/Tet^+^CD8^+^ T cells [23]. In addition, TPMs originating from A10-PPARα mice presented the greatest CD8^+^ T-cell response via enhanced antigen presentation. The antigen-presenting activities of both WT and A10-PPARα-Cre TPMs were equivalent and less than those observed in A10-PPARα cells (Fig. 5C). Thus, as investigated in several different ways, the selective depletion of PPARα in macrophages consistently downregulates the immune response of A10-PPARα mice to a functional level equivalent to that of WT mice.

We also separately analyzed the background effect of the LysM-Cre allele on the phenotype of ACE10/10 mice via ACE10/10-Cre mice. Analysis of B16-F10 tumor size in these mice revealed no effect of LysM-Cre expression; these mice developed tumors equivalent in size to those in ACE10/10 mice (Supplementary Fig. 2D). We also measured macrophage function and the frequency of TRP-2/Tet^+^CD8^+^ T cells in the tumor microenvironment, none of which differed between ACE10/10-Cre mice and ACE10/10 mice (Supplementary Fig. 2E–I). These results indicate that the lack of PPARα in A10-PPARα-Cre mice, not the unusual Cre recombinase effects or the lack of a single allele of the *Lyz2* gene, affects the impaired antitumor response.

### Reduced bactericidal effect on A10-PPARα-Cre mice

**Fig. 6:**
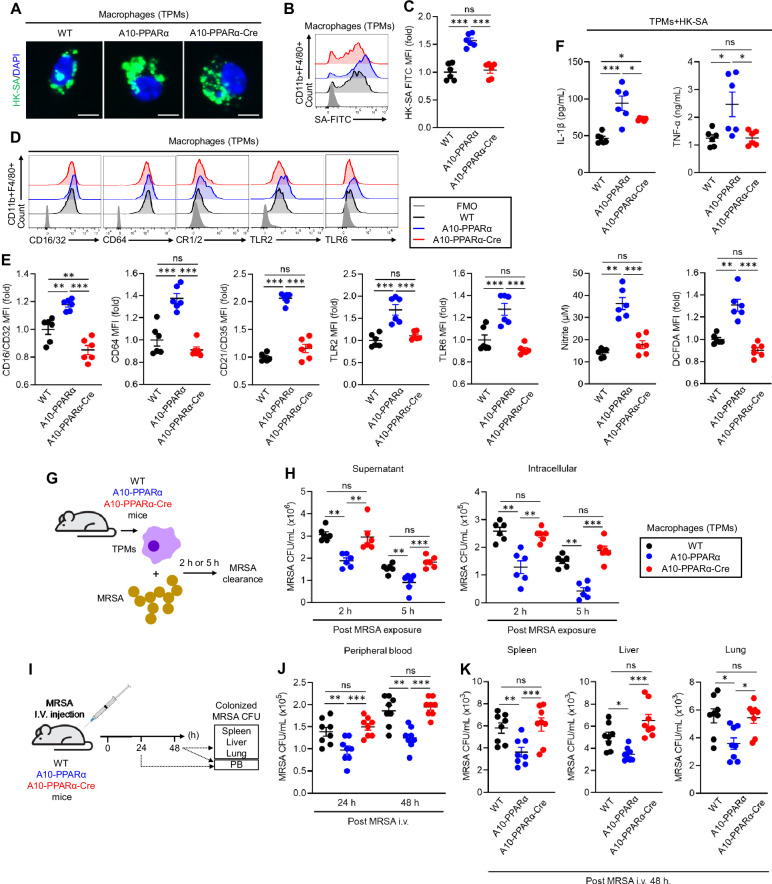
PPARα depletion impairs the antibacterial immune response of A10-PPARα-Cre mice. **A**–**C** In vitro phagocytosis. TPMs were incubated with FITC-labeled heat-killed *S. aureus* (HK-SA-FITC) at 37 °C for 2 h. Bacterial phagocytosis was analyzed via fluorescence microscopy and flow cytometry. **A** Representative fluorescence microscopy images of the incorporated HK-SA-FITC (green spots) in the TPM (bar = 10 μm). Representative histograms (**B**) and MFI values (fold change) (**C**) of incorporated HK-SA-FITC signals in TPMs. **D**, **E** Cell surface receptor expression in TPMs. Representative histograms (**D**) and MFI values (fold change) (**E**) of CD16/CD32, CD64, CR1/2, TLR2, and TLR6 are shown. **F** In vitro TPM stimulation assay. TPMs were stimulated with HK-SA at 37 °C for 24 h, and the concentrations of IL-1β, TNF-α, and nitrite in the culture medium were measured via ELISA and the Griess assay. ROS production in TPMs was measured by flow cytometry with DCFDA staining. **G**, **H** In vitro MRSA killing. **G** In vitro bactericidal activity assay. TPMs (1.0 × 10^6^/mL) were incubated with MRSA (1.0 × 10^7^ CFU/mL, MOI = 1:10) for 2 h or 5 h, and the number of live MRSA in the supernatant and within the TPM was quantitated as colony-forming units (CFUs). The bacterial CFUs in the supernatant and intracellular mixture are shown in (**H**). **I**–**K** In vivo MRSA infection. **I** In vivo bactericidal activity assay. The mice received an i.v. injection of live MRSA (100 μL of 1.0 × 10^9^ CFU/mL in PBS). After 24 h or 48 h, the number of MRSA CFUs in the peripheral blood (PB) was measured (**J**). The number of MRSA CFUs in the spleen, liver, and lung was also measured at 48 h (per 100 mg of tissue) (**K**). All MFI values are represented as fold changes (the average value of WT was used as 1). The cumulative data are shown as the means ± SEMs of six or eight samples from two or three independent experiments. One-way ANOVA was used to analyze the data for significant differences. *p < 0.05, **p < 0.01 and ***p < 0.01. ns not significant

To investigate an immune challenge different from B16-F10 melanoma, we studied the ability of the three groups of mice to respond to the challenge with methicillin-resistant *Staphylococcus aureus* (MRSA). In the first experiment, we performed an in vitro phagocytosis assay using TPMs from the three groups of mice. The TPMs were incubated with fluorescein isothiocyanate (FITC)-labeled heat-killed *S. aureus* (HK-SA-FITC) for 2 h, after which phagocytic activity was assessed via flow cytometry. This pattern was typical of previous results in that A10-PPARα TPMs captured more bacteria than WT or A10-PPARα-Cre TPMs did (Fig. 6A–C). Furthermore, TPMs from WT, A10-PPARα, and A10-PPARα-Cre mice presented significant differences in surface receptor expression, which is indispensable for the recognition and capture of gram-positive bacteria such as MRSA [24–26]. Compared with those from WT and A10-PPARα-Cre cells, TPM from A10-PPARα mice expressed increased basal levels of CD16/CD32 (FcγRII/III), CD64 (FcγRI), CD21/CD35 (complement receptor (CR) 1/2), Toll-like receptor (TLR) 2, and TLR6 (Fig. 6D, E). WT and A10-PPARα-Cre TPMs expressed equivalent levels of these surface receptors, except for CD16/CD32, where cells from A10-PPARα-Cre mice expressed significantly lower levels than WT cells did. Cytokine and chemical mediator production was measured in TPMs exposed to HK-SAs. TPMs from the three groups of mice incubated with HK-SA for 24 h presented a consistent pattern in which A10-PPARα cells produced greater amounts of IL-1β, TNF-α, ROS, and nitrite. The TPMs from the A10-PPARα-Cre mice produced smaller amounts of these substances, equivalent to WT cells, except for IL-1β, where the level of expression in the A10-PPARα-Cre TPMs was intermediate between that of the WT and the A10-PPARα mice (Fig. 6F).

To assess the ability of live bacteria to kill macrophages directly, TPMs from the three groups of mice were mixed in vitro with MRSA for 2 or 5 h, the macrophages were then separated by centrifugation, and the number of colony-forming units (CFUs) in the supernatant and in the lysed macrophages was assessed (Fig. 6G). The observed pattern was again characterized by TPMs from A10-PPARα mice being the most efficient at killing MRSA, whereas cells from the other two groups were both equivalent and less effective at killing bacteria (Fig. 6H).

As a final experiment to measure in vivo resistance to bacterial infection, mice from the three groups were injected i.v. with MRSA and bacterial CFUs were measured in the peripheral blood (PB) at 24 h and in the PB, liver, spleen, and lung at 48 h. For the solid organs, tissue was homogenized in PBS, and bacteria were measured per 100 mg of tissue (Fig. 6I). The results of these assays were similar to what was previously reported: samples from A10-PPARα mice presented significantly fewer MRSA CFUs than the samples from the other two groups did. However, the bactericidal effects on WT and A10-PPARα-Cre mice were not significantly different. Finally, the TPMs from ACE10/10-Cre mice showed similar activity against bacteria as did those from ACE10/10 mice (Supplementary Fig. 2J, K). Thus, macrophage PPARα depletion in A10-PPARα-Cre mice critically impairs antibacterial responses.

### PPARα is a key regulator of ACE-dependent functional upregulation in human macrophage-like cells

**Fig. 7:**
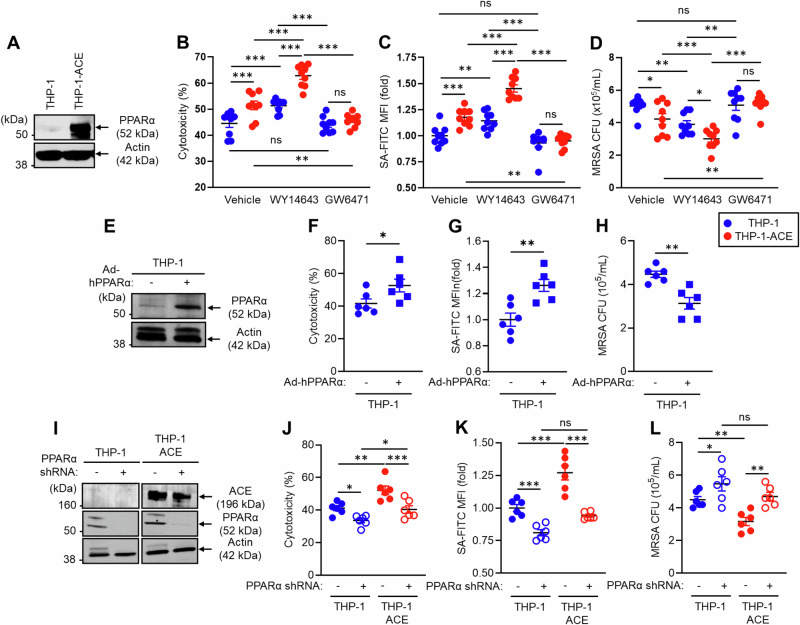
PPARα regulates the ACE-mediated functional behavior of human macrophage-like cells. THP-1 and THP-1-ACE cells were differentiated into macrophage-like cells by treatment with 20 ng/mL PMA for 72 h. **A** Western blot (WB) image of ACE expression. **B** In vitro tumor killing. BT549 cells and macrophage-like cells were mixed at a 1:1 ratio and incubated at 37 °C for 24 h. LDH concentrations in the culture media were measured to calculate the degree of killing of the tumor cells. **C** In vitro phagocytosis. Macrophage-like cells were incubated with FITC-labeled *S. aureus* (SA-FITC) for 2 h at 37 °C, after which bacterial phagocytosis was quantified via flow cytometry. **D** In vitro MRSA killing. Macrophage-like cells were incubated with MRSA (MOI = 1:30) at 37 °C for 5 h. The number of live intracellular MRSA was then determined via CFU analysis. In this assay, the cells were also treated with vehicle (DMSO), WY 146743 (a PPARα agonist), or GW6471 (a PPARα antagonist). **E** WB image of PPARα expression in THP-1 cells. Human PPARα was overexpressed in THP-1 cells via adenovirus transduction. THP-1 cells and PPARα-overexpressing (OE) THP-1 cells were used to measure in vitro tumor killing (**F**), in vitro phagocytosis (**G**), and in vitro MRSA killing (**H**). **I** WB image of ACE and PPARα expression in PPARα-knockdown (KD) THP-1 or THP-1-ACE cells. PPARα mRNA expression was silenced via shRNA. These cells were used to measure in vitro tumor killing (**J**), in vitro phagocytosis (**K**), and in vitro MRSA killing (**L**). All MFI values are represented as fold changes (the average value of the control was used as 1). The cumulative data are shown as the means ± SEMs of six to nine samples from two or three independent experiments. Student’s *t*-test or one-way ANOVA was used to analyze the data for significant differences. *p < 0.05, **p < 0.01 and ***p < 0.01. ns not significant

To investigate the role of PPARα in ACE-mediated functional enhancement of human myeloid cells, we performed in vitro functional assays using the human monocytic cell lines THP-1 and THP-1-ACE [9]. THP-1-ACE cells were genetically altered to stably overexpress catalytically active human ACE (Fig. 7A) [9]. These cells were used for assays after differentiation into macrophage-like cells [9]. Additionally, the assays were performed in the presence or absence of the PPARα agonist WY14643 or the antagonist GW6741 [9, 27, 28]. An in vitro tumor-killing assay revealed that, compared with THP-1 cells, THP-1-ACE had significantly increased cytotoxicity against BT549 cells, a human breast cancer cell line. WY14643 treatment increased the activity of both THP-1 and THP-1-ACE cells and increased the difference between the two groups. In contrast, this difference was abolished by the PPARα antagonist GW6471 (Fig. 7B). Compared with that of THP-1 cells, the phagocytic activity of THP-1-ACE cells against *S. aureus* was also significantly greater. Moreover, the WY14643 treatment augmented the phagocytic activity of both cell types, and the difference between the two groups was increased by this treatment. GW6471 decreased the phagocytic activity of THP-1-ACE cells, which was equivalent to that of THP-1 cells (Fig. 7C). We performed an in vitro bacterial killing assay by incubating the cells with MRSA for 5 h, after which the bacterial CFUs were measured to estimate the bactericidal effects. This assay revealed that the bactericidal effect of THP-1-ACE was significantly greater than that of THP-1. The effect was further increased by WY14643 and impaired by GW6471 (Fig. 7D).

To investigate the contribution of increased PPARα to human macrophage immune responses, we generated PPARα-overexpressing THP-1 cells via an adenoviral vector transduction system. Cellular PPARα protein expression was increased more than 10-fold by this approach (Fig. 7E). The tumor-killing effect, bacterial phagocytosis, and bactericidal effect were significantly greater in PPARα-overexpressing THP-1 macrophages than in control cells (Fig. 7F–H). In contrast, PPARα knockdown (KD) impaired the function of human macrophages. Specifically, PPARα mRNA expression was silenced by a lentiviral vector in which short hairpin RNA (shRNA) was introduced into THP-1 and THP-1-ACE cells (Fig. 7I). PPARα protein expression was strongly decreased by the shRNA in both the THP-1 and THP-1-ACE cells, but the shRNA did not affect ACE expression. Reduced PPARα protein expression decreased the tumor-killing effect of THP-1 and THP-1-ACE macrophages (Fig. 7J). The ability of bacteria to deal with bacteria was also impaired by PPARα silencing, resulting in decreased bacterial phagocytosis and clearance in both THP-1 and THP-1-ACE macrophages compared with control cells (Fig. 7K, L). Decreased PPARα expression was associated with similar levels of immune activity between THP-1 and THP-1-ACE macrophages, indicating the important role of this protein in the function of human macrophages and thus complementing our studies in mice. Thus, overexpression of ACE enhances human macrophage function, similar to observations in mouse macrophages, and PPARα has consistently emerged as a necessary factor regulating this functional modification.

### Selective activation of PPARα enhances antitumor immunity in a physiological environment, and this effect is amplified by myeloid lineage-specific ACE overexpression

**Fig. 8:**
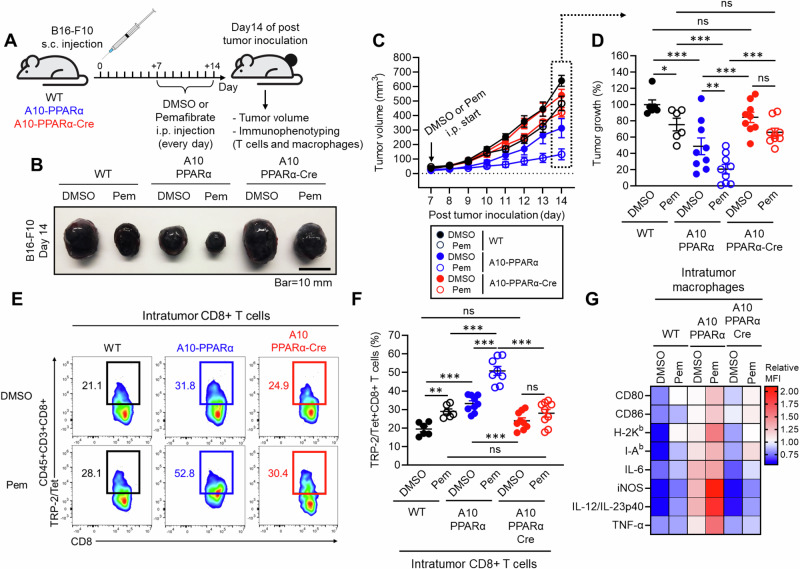
Selective PPARα activation enhances antitumor immunity. **A** Effect of a selective PPARα agonist in a murine melanoma model. The mice received a s.c. injection of B16-F10 cells on the same day. Beginning on day 7, DMSO (control) or 10 mg/kg pemafibrate was administered daily for 7 days. On day 14, the tumor volume was measured, and intratumor (IT) CD8^+^ T cells and macrophages were studied via flow cytometry. **B** Representative pictures of tumors. **C** Measurement of tumor volume. **D** Percentage of control tumor volume where tumors in the DMSO-treated WT group were set as 100%. **E** Representative plots of TRP-2/Tet^+^CD8^+^ T cells in tumors. **F** TRP-2/Tet^+^CD8^+^ T cells as a percentage of all CD8^+^ T cells in tumors. **G** Heatmap characterization of IT macrophages for receptors and cytokines important for the antitumor response. The data are shown as the means ± SEMs of six to nine tumor samples (one sample/mouse) from three independent experiments. One-way ANOVA was used to analyze the data for significant differences. *p < 0.05, **p < 0.01 and ***p < 0.01. ns not significant

Finally, we tested whether selective PPARα function activation enhances the immune system. To this end, we employed a murine B16-F10 melanoma model with or without PPARα agonist treatment. Beginning one week after tumor inoculation and continuing for a total of 7 days, the mice received a daily intraperitoneal (i.p.) injection of either DMSO (control) or pemafibrate (Pem), a highly selective PPARα agonist used in humans for the treatment of metabolic syndrome [29, 30]. Tumor growth was monitored from the start of drug treatment, and immune phenotyping of T cells and macrophages was performed after the mice were sacrificed on day 14 (Fig. 8A). Compared with the control treatment, pemafibrate significantly reduced the tumor volume in WT mice (DMSO: 640.21 ± 30.90 mm³ vs Pem: 483.31 ± 40.51 mm³, mean reduction of 24.5%). Similarly, A10-PPARα-treated mice presented a significant reduction in tumor volume with pemafibrate compared with the A10-PPARα control group (DMSO: 312.69 ± 65.92 mm³ vs Pem: 131.94 ± 37.59 mm³, mean reduction of 57.8%), with the effect on tumor suppression being more pronounced than that in WT mice. Interestingly, A10-PPARα-Cre mice also showed an effect of pemafibrate on tumor suppression (DMSO: 540.44 ± 40.66 mm³ vs Pem: 401.25 ± 32.35 mm³, mean reduction of 25.8%), similar to that observed in WT mice (Fig. 8B–D).

We also assessed the immune activity of CD8^+^ T cells and macrophages in the tumor microenvironment (TME). Compared with DMSO treatment, Pemafibrate treatment significantly increased the frequency of tumor antigen-specific TRP-2/Tet^+^CD8^+^ T cells in WT mice. This increase was much more pronounced in A10-PPARα mice, with more than 50% of the CD8^+^ T cells in the tumor microenvironment being TRP-2/Tet^+^ for their T-cell receptor. A10-PPARα-Cre mice presented a slight increase in the frequency of TRP-2/Tet^+^CD8^+^ T cells following pemafibrate compared with vehicle (DMSO), but this difference was not statistically significant (Fig. 8E, F). Additionally, we found that macrophage activity was increased by pemafibrate treatment, and this effect was detected in A10-PPARα mice but not in A10-PPARα-Cre mice (Fig. 8G). Thus, selective PPARα activation in macrophages contributes to the enhancement of CD8^+^ T-cell-mediated antitumor activity.

## Discussion

Discovering ways to increase the immune response is important from the perspective of understanding immune system function and from a practical desire to increase immunity against tumors and serious infections. Such increased immunity is observed in ACE10/10 mice, in which the ACE gene is under the control of the myeloid lineage-specific colony-stimulating factor 1 receptor (*c-fms*) promoter, resulting in increased ACE protein production in monocytes and macrophages. Several previous studies revealed that ACE10/10 mice have highly effective immune responses to a variety of disease conditions, such as cancers, infectious diseases, atherosclerosis, and Alzheimer’s disease (AD) [4–12]. Despite having an immune response that is far more effective than WT, ACE10/10 mice show no evidence of immunological abnormalities, such as chronic inflammatory and autoimmune conditions. The phenotypes of ACE10/10 mice are directly due to the increased catalytic activity of ACE, as ACE inhibitors reduce the modified immune responses of these mice to those of WT mice treated similarly [5–12]. Furthermore, a different line of mice with increased ACE expression in a granulocyte-specific manner termed NeuACE mice, demonstrated a significantly increased neutrophil antibactericidal effect, as proven by challenge with several different pathogenic bacteria [31, 32]. While studies of the immune function of ACE in humans are far less extensive than studies in mice are, there is evidence that blocking ACE activity also reduces human neutrophil activity against bacteria [32]. Thus, it is potentially very important to understand specifically how ACE activity enhances myeloid cell immune function.

Recently, our studies revealed that ACE activity markedly changes the metabolic characteristics of myeloid cells overexpressing ACE [9, 10, 12]. The overexpression of ACE significantly increased the activity of mitochondrial oxidative metabolism, particularly the utilization of long-chain fatty acids (LCFAs), resulting in increased intracellular ATP levels in macrophages. These metabolic alterations are so striking that they ineluctably lead to the hypothesis that this is what underpins the increased functionality of macrophages expressing increased ACE.

PPARα was first discovered in 1990 as a ligand-receptor capable of inducing hepatic peroxisome proliferation [33]. Subsequent studies of PPARα, specifically in the liver and hepatocytes, established that this transcription factor is one of the central regulators of lipid metabolism [18, 34]. Among the three PPARs known (α, γ, and β/δ), macrophages exhibit higher expression of PPARγ than PPARα [35, 36], and perhaps because of this, there has not been extensive study of the functional effects of PPARα in macrophages. However, some immunological aspects of PPARα have been studied in different cell types and systems. Studies of PPARα global knockout (KO) mice, which show an increase in cytokine expression in some instances, have suggested an anti-inflammatory role of this protein [37, 38]. In another study, PPARα-KO mice presented increased IL-17A^+^CD4^+^ T-cell (Th17) generation, resulting in increased pathological features of murine experimental autoimmune encephalomyelitis (EAE) [39]. Another study reported that inhibition of PPARα activity restored the antitumor effect of dendritic cells [40].

Our interest in PPARα came from the finding that this protein was elevated in ACE10/10 macrophages under both basal conditions and after exposure to lipids and that several known targets of PPARα, such as CPT1A, CPT1B, CPT2, acyl-CoA dehydrogenase long chain (ACADL), CD36, and ATP-binding cassette transporter 1 (ABCA1), were elevated in these same cells [9, 10]. In contrast, no such increase was noted for PPARγ or PPARδ. Lipid tracing via ^13^C-OA revealed that ACE10/10 macrophages metabolize lipids far more effectively than do WT cells [9]. Furthermore, the increased oxidative metabolism and increased ATP levels in these cells appeared to support increased macrophage functional capacity [9, 10, 12]. The upregulated gene expression via activated PPARα promotes fatty acid β-oxidation within mitochondria, and the resulting increase in ATP led us to create A10-PPARα-Cre mice to precisely evaluate the role of this master lipid regulator in the exceptional immune response of ACE10/10 mice. Indeed, one of the critical findings of our study is that ATP levels in A10-PPARα-Cre macrophages were reduced to levels present in WT cells. Whether ATP is the full explanation or, more likely, that the global change in metabolism present in ACE10/10 macrophages underpins the increased function remains to be determined. However, increased PPARα clearly has an indispensable role in enhancing macrophage immune activity in ACE10/10 mice, as proven by gene expression profiles, metabolic characterization, and two different types of immune challenge, tumor and bacterial infection, in this study.

Our data raises several questions. First, we do not know exactly how ACE elevates PPARα. What we know from many previous studies is that ACE must be catalytic to stimulate an increased myeloid immune response. In fact, while ACE has two catalytic domains, the catalytic activity of the ACE C-domain is critical [41]. However, we do not yet know the exact peptide that markedly affects macrophage function.

Another question is the role of PPARα in the immune response of *WT* macrophages. To address this, we performed in vitro experiments using the human monocytic cell line THP-1. PPARα stimulation via the PPARα agonist WY14643 increased THP-1 macrophage activity. The reduction in PPARα activity caused by the antagonist GW6471 had minimal effects on WT cells, but the reduction in PPARα activity caused by RNA knockdown resulted in decreased cytotoxicity and bacterial killing compared with those in control cells. Thus, these data suggest a role for PPARα in the immune function of WT macrophages.

Our data raise a third much more important question: does forcing increased PPARα activity in macrophages lead to increased immune function? This effect was tested in vitro by treating THP-1 and THP-1-ACE cells with the PPARα-selective ligand WY14643. As noted above, WY14643 increased the immune function of both cell types in vitro, and, as shown in Fig. 8, the use of pemafibrate in vivo also induced reduced tumor growth in both WT and ACE10/10 mice. Compared with WY14643 and other fibrates, pemafibrate has increased specificity and affinity for PPARα. The effect of this drug in vivo was striking: tumor reduction in both WT and A10-PPARα mice had a somewhat synergistic effect with the increased ACE present in A10-PPARα mice. Pemafibrate enhanced antitumor immunity with an increased frequency of tumor antigen-specific CD8^+^ T cells in the tumor. While A10-PPARα-Cre mice treated with pemafibrate tended toward smaller tumors, this trend did not reach significance, a result that might have been predicted given the reduction in macrophage PPARα in these mice. Thus, this experiment again indicates the important role of macrophage PPARα levels and activity in the immune response. In summary, previous studies of ACE10/10 mice have reported that a remarkable increase in mouse immune function is achieved by increasing the expression of ACE in macrophages. An indispensable component of the elevated immune response is macrophage PPARα expression and activity.

## Materials and methods

### Reagents and antibodies

Oleic acid (OA) and bovine serum albumin (BSA, fraction V, fatty acid-free) were purchased from Roche (Basel, Switzerland), and collagenase type II, phorbol 12-myristate 13-acetate (PMA) and ionomycin were purchased from MilliporeSigma (Burlington, MA, USA). 2′,7′-Dichlorodihydrofluorescein diacetate (H_2_DCFDA), MitoSOX™, 10× red blood cell (RBC) lysis buffer (multiple species), fluorescein isothiocyanate (FITC)-labeled *Escherichia coli* (*E. coli*) K-12 strain, and FITC-labeled heat-killed *Staphylococcus aureus* (HK-SA) were purchased from Thermo Fisher Scientific (Waltham, MA, USA). Macrophage colony-stimulating factor (M-CSF) was purchased from PeproTech (Westlake Village, CA, USA). Heat-killed *Staphylococcus aureus* (HK-SA) was purchased from InvivoGen (San Diego, CA, USA). CellTiter-Glo® 2.0 was purchased from Promega (Madison, WI, USA). Lipi-Deep Red and the lipid peroxidation probe BDP 581/591 C11 were purchased from Dojindo Laboratories (Kumamoto, Japan). The monoclonal antibodies (mAbs) and tetramers used for flow cytometry are shown in Supplementary Table 1. The Abs used for western blotting are shown in Supplementary Tables 3 and 4.

### Mice

To generate ACE10/10-PPARα-floxed (ACE10/10-PPARα^flox/flox^) mice, WT PPARα-floxed (PPARα^flox/flox^) mice [originally made by Dr. Walter Wahli [35], University of Lausanne] were obtained from Dr. Mingyu Liang, Medical College of Wisconsin. These mice were crossed with ACE10/10 mice. The ACE10/10-PPARα^flox/flox^ mice were further crossed with heterozygous B6.129P2-*Lyz2*^*tm1(cre)Ifo*^/J (Jackson Labs strain #4781) [19] that was a gift from Dr. Gislaine Martins, Cedars-Sinai Medical Center. Eventually, we obtained ACE10/10-PPARα^flox/flox^-LysM-Cre^−^^/−^ (A10-PPARα) and ACE10/10-PPARα^flox/flox^-LysM-Cre^+/−^ (A10-PPARα-Cre) mice. The genetic design of the mice is shown in Fig. 1A. The genotype of each mouse was determined via PCR via the primers shown in Supplementary Table 1. All the mice were bred with 12 h day/night cycles and were allowed free access to food and water. Sex- and age-matched adult mice (8–16 weeks) were used for each experiment. All animal experimental protocols were reviewed and approved by the Animal Welfare Committee Cedars-Sinai Medical Center (#8780).

### Murine melanoma model

A murine melanoma model was established by following the methods reported in a previous publication [6, 42]. Briefly, B16-F10 cells (1.0 × 10^6^ cells in 100 µL of PBS) were subcutaneously (s.c.) injected into the back skin of the mice via a 1 mL syringe with a 25 G needle. The tumor volume was measured on day 14 via the following formula: *V* = (*L* × *W*^2^) × 0.52 (*V*: tumor volume, *L* > *W*). Finally, the mice were sacrificed on day 14 for immunological analysis. The excised tumors were studied for intratumor (IT) macrophages and T-cell analysis. For administration of the PPARα agonist pemafibrate, the mice first received s.c. injection of B16-F10 cells (1.0 × 10^6^ cells in 100 µL of PBS), and then, the mice received daily intraperitoneal (i.p.) injection of DMSO or pemafibrate (10 mg/kg) (both diluted with PBS) from day 7 posttumor inoculation through day 13 (for a total of 7 days). The tumor volumes were measured every day during the DMSO- or pemafibrate-administered periods. The mice were sacrificed on day 14 posttumor inoculation, and the cells isolated from the tumors were subjected to flow cytometry analysis [43].

### Preparation of thioglycolate-elicited peritoneal macrophages (TPMs)

The mice received an intraperitoneal (i.p.) injection of 3 mL of thioglycolate, and the infiltrated leukocytes were harvested from the peritoneum on day 4 (between 84 and 96 h) postinjection. The cells were seeded into 100 mm cell culture dishes in complete RPMI medium (RPMI 1640 supplemented with 10% fetal bovine serum (FBS), 100 U/mL penicillin, and 100 µg/mL streptomycin) and incubated at 37 °C for 3 h. The adherent cells, which were enriched with TPMs, were washed with PBS three times and then detached by gently scraping in PBS/2% FBS. The purity of the TPMs was assessed via flow cytometry. The samples with CD11b^+^F4/80^+^ > 90% were used for subsequent experiments.

### Gene expression profiling

Bulk RNA sequencing was performed on total RNA isolated from thioglycolate-elicited peritoneal macrophages from WT, A10-PPARα, and A10-PPARα-Cre mice (*n* = 3 in each group). Alternatively, some of these macrophages were cultured in RPMI 1640 medium supplemented with 1% BSA (fraction V, fatty acid-free), 100 U/mL penicillin, 100 µg/mL streptomycin, and recombinant murine macrophage colony-stimulating factor (rmM-CSF, 10 ng/mL) containing either vehicle (ethanol) or OA (200 µM) at 37 °C for 36 h. Total RNA was isolated by using the RNeasy Plus Mini Kit (QIAGEN, Hilden, Germany) following the product manual. The RNA samples with A260/280 values ranging from 1.8 to 2.0 were subjected to RNA sequencing. RNA sequencing was performed by BGI (https://www.bgi.com/global). The TPM (transcripts per million) values were converted to log2(TPM + 1) values and used for MA plot generation, pathway identification, and heatmap generation. In the pathway analysis, *p*-values and fold changes (based on mean values of log2(TPM + 1) for each group) were calculated in the following comparisons: A10-PPARα-Cre vs. WT and A10-PPARα-Cre vs. WT. These values were used in Ingenuity Pathway Analysis (IPA; QIAGEN, Hilden, Germany) to identify functional pathways exhibiting differences in gene expression between the comparisons. Genes with statistically significant *p*-values (*p* < 0.05) were used in IPA for pathway identification and to calculate the Rich ratio. The figures present identified pathways in which the Rich ratio for A10-PPARα-Cre vs. WT is either greater than 1.2-fold or less than 0.8-fold greater than that of A10-PPARα-Cre vs. WT. Additionally, pathways with a Rich ratio of 0 for A10-PPARα-Cre vs. WT are also shown. To generate heatmaps, gene sets associated with metabolism and the immune response were identified via the Kyoto Encyclopedia of Genes and Genomes (KEGG) database (https://www.genome.jp/kegg/). The mean log2(TPM + 1) values of the WT were compared to those of A10-PPARα or A10-PPARα-Cre independently, and the 20 genes with the greatest increase in expression from the WT for each comparison were extracted to generate heatmaps. z scores, calculated from log2(TPM + 1) values, were used to visualize gene expression differences in the heatmaps.

### Flow cytometry

Flow cytometry analysis was performed by using a Cytek NL-3000 (Cytek Bioscience, Fremont, CA, USA). For extracellular marker staining, the samples were stained with a fluorochrome-conjugated mAb or tetramer in the presence of an anti-CD16/CD32 mAb to block the Fc gamma receptor (FcγR) II/III at 4 °C for 30 min. Intracellular staining was performed via the BD Cytofix/Cytoperm™ Fixation/Permeabilization Kit (BD Bioscience, Franklin Lakes, NJ, USA). For intracellular staining, the extracellularly stained cells were fixed at 4 °C for 20 min, followed by staining of the intracellular targets at 4 °C for 30 min. The samples studied for cytokine detection in T cells were stimulated with PMA (100 ng/mL) and ionomycin (250 ng/mL) in the presence of GolgiStop^TM^ (1 μg/mL, BD Bioscience) at 37 °C for 5 h. The mAbs and tetramers used in the flow cytometry analyses are described in Supplementary Table 2. The samples were analyzed via flow cytometry following the gating strategies represented in Supplementary Fig. 11. The data were analyzed with FlowJo^TM^ v10 (BD Bioscience).

### Western blotting

For protein isolation, the cells were treated with RIPA buffer supplemented with a protease inhibitor cocktail (Thermo Fisher Scientific, 1:100 dilution) and ethylenediaminetetraacetic acid (EDTA, 5 mM at the final concentration). The samples were sonicated at 4 °C and stored at −20 °C overnight, followed by centrifugation at 12,000 × *g* for 15 min. The supernatant was collected as an extracted protein sample. The protein concentration was determined via a bicinchoninic acid (BCA) assay. For loading sample preparation, the proteins were diluted with 5× sodium dodecyl sulfate (SDS) sample buffer followed by boiling at 95 °C for 5 min. The proteins were separated via SDS‒polyacrylamide gel electrophoresis (SDS‒PAGE) in 4‒12% gradient gels (Thermo Fisher Scientific) followed by transfer onto polyvinylidene fluoride (PVDF) membranes. The membranes were blocked in blocking buffer (5% milk in Tris-buffered saline containing 0.1% Tween 20 (TBST)) at RT for 60 min and then treated with primary Ab (diluted with 1% milk in TBST) at 4 °C overnight. After being washed with TBST, the membranes were treated with secondary Ab (diluted with 1% milk in TBST) at RT for 60 min. The protein bands were visualized via an Odyssey CLx Infrared Imaging System (LI-COR, St., Lincoln, NE, USA). The antibodies used for immunoblotting are shown in Supplementary Tables 3 and 4.

### Reverse transcription-quantitative polymerase chain reaction (RT‒qPCR)

TPMs (1.0 × 10^6^/mL) were treated with OA (200 µM) in RPMI 1640 medium supplemented with 1% BSA (fraction V, fatty acid-free), 100 U/mL penicillin, 100 µg/mL streptomycin, and rmM-CSF (10 ng/mL) at 37 °C for 16 h. Total RNA was subsequently isolated from the cultured TPMs via TRIzol (Thermo Fisher Scientific), and the concentration and purity were subsequently measured via a NanoDrop 2000c (Thermo Fisher Scientific). Total RNA (500 ng) was reverse transcribed into complementary DNA (cDNA) via Superscript II (Applied Biosystems, Waltham, MA, USA). The cDNA was used for quantitative PCR via a 7500 real-time PCR system (Applied Biosystems) and iTaq Universal SYBR Green Supermix (Bio-Rad Laboratories, Hercules, CA, USA). The *Gapdh* gene was used as an internal control. The difference in mRNA expression was quantified via the ∆Ct method. The sequences of primers used in the analysis are shown in Supplementary Table 5.

### Cell isolation from tumors

The tumors were excised from the back skin of the mice and briefly washed with PBS. The tumor was chopped with scissors in the digestion medium (RPMI complete medium supplemented with collagenase type II (1 mg/mL)) followed by incubation with shaking at 37 °C for 15 min. Then, the sample was passed through a 70 µm cell strainer, and the tissue fragments were mechanically crushed on the cell strainer. The (IT) intratumor cells were collected by centrifugation at 300 × *g* for 5 min, after which the cells were washed with complete RPMI 1640 medium. Finally, the cells were collected by centrifugation at 300 × *g* for 5 min and resuspended in a complete RPMI medium. The cell mixture was then stained for flow cytometry, with the specific determination of macrophages and T cells dependent on typical cell surface markers (see the “Flow cytometry” section).

### Primary cell isolation

Spleen and lymph nodes (LNs) were mechanically crushed on a 70 µm cell strainer in a complete RPMI medium, and then the cells were collected by centrifugation at 300 × *g* for 5 min. The cells were treated with red blood cell (RBC) lysis buffer at RT for 10 min and then washed with a complete RPMI medium. After centrifugation at 300 × *g* for 5 min, the precipitated cells were used as splenocytes and LN-isolated cells, respectively. Peripheral blood (PB) was treated with RBC lysis buffer at RT for 10 min, followed by washing with PBS. After centrifugation at 300 × *g* for 5 min, the precipitated cells were used as PB leukocytes. Bone marrow (BM) cells were isolated from femurs and tibias. The cells were flushed from the bones via a 10 mL syringe with a 27 G needle in a complete RPMI medium. The cells were collected by centrifugation at 300 × *g* for 5 min and then treated with RBC lysis buffer at RT for 10 min. After washing with PBS, the cells were collected by centrifugation at 300 *×* *g* for 5 min. The precipitated cells were used as BM-derived cells. Hepatocytes and hepatic leukocytes were isolated from the whole liver. The liver was excised from a PBS-perfused mouse and chopped and mechanically crushed on a 70 µm cell strainer in a complete RPMI medium. The cells were collected by centrifugation at 300 × *g* for 5 min, and the precipitated cells were resuspended in 35% Percoll (diluted with PBS) for density gradient separation by centrifugation at 600 *×* *g* for 20 min without rapid acceleration or external braking. After centrifugation, the top layer was collected as hepatocytes, and the precipitated cells were further treated with RBC lysis buffer at RT for 10 min. The samples were washed with a complete RPMI medium and centrifuged at 300 × *g* for 5 min. The precipitated cells were used as hepatic leukocytes (mononuclear cells; MNCs).

### Metabolic assay of macrophages

TPMs (1.0 × 10^6^/mL) were cultured with vehicle (ethanol) or OA (200 µM) in RPMI 1640 medium supplemented with 1% BSA (fatty acid-free), 100 U/mL penicillin, 100 µg/mL streptomycin and rmM-CSF (10 ng/mL) at 37 °C for 24 h. The cultured cells were used for subsequent analyses. For the adenosine triphosphate (ATP) assay, the TPMs were washed with PBS and then treated with CellTiter-Glo® 2.0 reagent at RT for 15 min, after which the luminescence intensity was read with a microplate reader (FLUOstar Omega, BGM LABTECH, Ortenberg, Germany). The ATP concentration was determined via the standard curve method. For detection of intracellular reactive oxygen species (ROS), the TPMs were treated with H_2_DCFDA (5 µM) at 37 °C for 60 min, followed by flow cytometry analysis. Mitochondrial ROS (mtROS) were detected by staining with MitoSOX™ (1 µM) at 37 °C for 60 min, followed by flow cytometry analysis. Alternatively, fresh TPMs were used for real-time cell metabolic assays via Seahorse cell analysis (Agilent, Santa Clara, CA, USA).

### Fatty acid uptake and consumption assay

For the fatty acid uptake assay, TPMs (1.0 × 10^6^/mL) were cultured with vehicle (ethanol) or OA (200 µM) in RPMI 1640 medium supplemented with 1% BSA (fatty acid-free), 100 U/mL penicillin, 100 µg/mL streptomycin and rmM-CSF (10 ng/mL) at 37 °C for 16 h. After washing with PBS, the TPMs were stained with Lipi-Deep Red (LDR, 1:200 dilution) at 37 °C for 2 h. The lipid droplet (LD) content in the TPMs was analyzed via flow cytometry. To investigate the fatty acid consumption rate, the TPMs were treated with OA following the protocol of the fatty acid uptake assay. After 16 h of fatty acid treatment, the TPMs were washed with PBS and maintained in a complete RPMI medium supplemented with rmM-CSF (10 ng/mL) until analysis. The TPMs were harvested at the indicated time points and stained with LDR (1:200) at 37 °C for 2 h, followed by flow cytometry analysis. The fatty acid consumption rate was calculated via the following formula: fatty acid consumption rate (%) equals (LDR MFI^0 h^ -LDR MFI^18 h^)/LDR MFI^0 h^ × 100. For fluorescence microscopic imaging, the TPMs (2.0 × 10^5^/mL) were seeded in a chamber slide and cultured following the same method described for OA uptake. After exposure to OA, some cells were stained with LDR immediately to measure baseline OA uptake (0 h), while other cells were cultured in a complete RPMI medium supplemented with rmM-CSF (10 ng/mL) for 18 h at 37 °C, followed by LDR staining to observe lipid consumption. LDR staining was performed following the same method used for flow cytometry analysis.

### Real-time metabolic assay

TPMs (1.5 × 10^5^/100 μL) were seeded on Cell-Tak (Corning, Corning, NY, USA)-coated Seahorse XF96 plates (Agilent Technologies, Santa Clara, CA, USA). All the analyses were performed in RPMI 1640 medium. Oligomycin (2 μM), FCCP (carbonyl cyanide-p-trifluoromethoxyphenylhydrazone, 500 nm), and rotenone (200 nM) with antimycin A (1 μM) were added to the wells, and the respiratory oxygen consumption rate was measured with a Seahorse XF96 analyzer (Agilent Technologies). The metabolic parameters were calculated via the following method as described in a previous report [12].

### Tumor-killing assay

TPMs (1.0 × 10^6^/mL) and B16-F10 cells (1.0 × 10^6^/mL) were mixed (at a ratio of 1:1) in complete RPMI medium at 37 °C for 24 h. Some cultures were prepared with TPMs or B16-F10 cells only as controls. The culture medium was harvested and stored at −80 °C until use. Macrophage tumor cytotoxicity assays were used to measure lactate dehydrogenase (LDH) release from B16-F10 cells. The abundance of released LDH was assessed by absorbance at 490 nm (background) and 680 nm (target) via the Pierce LDH Cytotoxicity Assay Kit (Thermo Fisher Scientific). The cytotoxicity was calculated via the following formula: % Cytotoxicity = (Coculture’s LDH activity – spontaneous B16-F10 death LDH activity)/(Maximum B16-F10 LDH activity – Spontaneous B16-F10 LDH activity) × 100.

### In vitro T-cell restimulation assay

The cells were isolated from the inguinal lymph nodes (iLNs) of B16-F10-inoculated mice (day 11). The iLN cells (3.0 × 10^6^/mL) were restimulated with TRP-2 (100 µg/mL) at 37 °C for 72 h. The IFN-γ concentration in the culture medium was measured via ELISA.

### In vitro antigen presentation assay

CD8^+^ T cells were isolated from the iLNs of B16-F10-inoculated WT mice (day 11). TPMs were prepared from WT, A10-PPARα, or A10-PPARα-Cre mice. CD8^+^ T cells (5.0 × 10^6^/mL) and TPMs (1.0 × 10^6^/mL) were cocultured in the presence of TRP-2 (100 μg/mL) at 37 °C for 24 h. CD69 expression and IFN-γ production in TRP-2/Tet^+^CD8^+^ T cells were analyzed via flow cytometry.

### Phagocytosis assay

TPMs (1.0 × 10^7^/mL) were incubated with FITC-labeled *S. aureus* (SA-FIC; 25 µg/mL) in a complete RPMI medium at 37 °C for 2 h. The macrophages were washed with PBS and analyzed by flow cytometry. The fluorescence signal (mean fluorescence intensity; MFI) originating from intracellularly incorporated bacteria was used to assess the phagocytic activity of the TPMs.

### Macrophage stimulation assay

TPMs (1.0 × 10^6^/mL) were seeded in 12- or 96-well plates with a complete RPMI medium. The cells were treated with vehicle (PBS) or HK-SA (1.0 × 10^7^ CFU/mL) at 37 °C for 24 h. After incubation, the culture medium was harvested and stored at −80 °C until use. The cytokine and nitric oxide (NO) concentrations in the cultured medium were measured via ELISA and the Griess assay, respectively. ROS production was measured by flow cytometry with H_2_DCFDA staining.

### Methicillin-resistant Staphylococcus aureus (MRSA) infection

MRSA (USA300) was obtained from the ATCC (Manassas, VA, USA) and cultured according to a method provided by the ATCC. Briefly, the frozen glycerol stock was thawed on ice, and the bacterial suspension was transferred to a tryptic soy broth (TSB) medium. The culture mixture was incubated at 37 °C overnight with shaking, after which the bacteria were further cultured in TSB medium at a 1:100 dilution at 37 °C for 6–8 h. The number of colony-forming units (CFUs) was determined via the standard curve method. The required number of bacteria was washed with PBS and collected by centrifugation at 10,000 × *g* for 1 min. The collected bacteria were resuspended in PBS or RPMI complete medium to adjust the concentration.

### In vitro MRSA killing assay

TPMs (1.0 × 10^6^/mL) were mixed with MRSA (1.0 × 10^7^ CFU/mL) in complete RPMI medium. The samples were incubated at 37 °C for 2 or 5 h. The samples were first centrifuged at 300 *×* *g* for 5 min, the supernatants were subsequently collected, and the precipitated cells were subsequently washed with PBS. The cells were again collected by centrifugation at 300 × *g* for 5 min and then treated with PBS/0.1% Triton X-100 at RT for 15 min to lyse the cells. The supernatant samples and cell lysates were used for the determination of MRSA CFUs by seeding on a TSB agar plate. The plates were incubated at 37 °C overnight, after which the number of MRSA CFUs in the samples was determined.

### MRSA infection

MRSA infection was performed following a method described in previous reports [31, 32]. Briefly, the MRSA was washed and resuspended in PBS at a concentration of 1.0 × 10^9^ CFU/mL. The mice received an intravenous (i.v.) injection of MRSA (100 µl of suspension) through the retroorbital sinus. Blood samples were collected at 24 h and 48 h post-infection. The tissue samples (spleen, liver, and lung) were collected at 48 h post-infection. Before organ extraction, the mice were perfused with PBS. The tissues were washed with PBS, chopped, and homogenized in PBS. The samples were serially diluted with PBS and seeded on TSB plates. The plates were incubated at 37 °C overnight, after which MRAS CFUs were determined in the samples.

### Cytokine and nitric oxide measurement

The cytokine concentration in each sample was measured with an ELISA kit (R&D Systems, Minneapolis, MN, USA) for each target. Nitric oxide (NO) concentrations in the samples were measured via the Griess Reagent System (Promega). All procedures followed the product manual.

### THP-1 cell culture, differentiation, and functional assays

THP-1 or THP-1-ACE cells were cultured in a complete RPMI medium and passaged every 5–6 days. For differentiation into macrophage-like cells, the cells were cultured in a complete RPMI medium supplemented with PMA (20 ng/mL) at 37 °C for 72 h. Differentiation status was confirmed by the increased expression of CD11b and CD14 compared with that in undifferentiated cells, as measured by flow cytometry analysis. To measure in vitro tumor killing, differentiated cells (1.0 × 10^6^/mL) were mixed with BT549 cells (1.0 × 10^6^/mL) at a ratio of 1:1 in a complete RPMI medium supplemented with vehicle (DMSO), WY14643 (10 μM), or GW6471 (10 μM) at 37 °C for 24 h. Some cultures were prepared with differentiated cells or BT549 cells only as controls. The tumor-killing activity of human macrophage-like cells was assessed by lactate dehydrogenase (LDH) release from BT549 cells and cytotoxicity was calculated via the method described above. For the in vitro *phagocytosis assay*, differentiated cells were initially cultured in RPMI complete medium supplemented with vehicle (DMSO), WY14643 (10 μM), or GW6471 (10 μM) at 37 °C for 16–18 h before being incubated with SA-FITC (50 μg/mL) at 37 °C for 2 h. The cells were then washed with PBS and analyzed by flow cytometry; the MFI value originating from intracellular bacteria was used to assess phagocytic activity. To measure the degree of intracellular killing of MRSA, differentiated cells were first treated with WY14643 or GW6471 as described above. Macrophage-like cells (1.0 × 10^6^/mL) were incubated with MRSA (3.0 × 10^7^ CFU/mL, MOI = 1:30) at 37 °C for 5 h and then washed and lysed to extract intracellular bacteria. Viable intracellular MRSA strains were then quantified by bacterial colony formation.

### Generation of PPARα-overexpressing cells

PPARα was overexpressed via an adenovirus vector-based transient overexpression system. The THP-1 cells were seeded at 5.0 × 10^5^/mL in RPMI complete medium in a 12-well plate and infected with Ad-human PPARα (at 1:25, 1:50, or 1:100 MOI; Vector Boiolab, Malvern, PA, USA). After 24 h of infection, the cells were washed and reseeded in 12-well plates in a fresh complete RPMI medium. The cells were harvested at 48 h postviral inoculation, after which PPARα expression was assessed via WB. The WB results revealed the highest PPARα expression at an MOI of 1:50; therefore, we selected this condition for subsequent experiments.

### Generation of PPARα-knockdown cells

PPARα-knockdown (KD) THP-1 or THP-1-ACE cells were generated via a lentiviral vector-based shRNA system [43]. To create a lentiviral vector, we first isolated a targeting plasmid (sh-NT or sh-PPARα) from the bacterial glycerol stock of *E. coli* provided by the MISSION shRNA system (MilliporeSigma). The bacterial glycerol stock was thawed on ice, and the bacteria were grown on LB agar plates supplemented with ampicillin (100 µg/mL) at 37 °C overnight. The independent colonies were precultured in 5 mL of LB media supplemented with ampicillin (100 µg/mL) at 37 °C for 6 h with shaking, after which the culture mixture was expanded to 50 mL and further incubated at 37 °C overnight. The plasmid was isolated by using a Midi Prep Kit (QIAGEN, Hilden, Germany). To create lentiviral vectors, HEK293T cells were seeded at 2.5 × 10^5^/mL in 12-well plates in complete DMEM, and the plasmid (500 ng) was transfected into the cells at 50–60% confluency via FuGeneHD (Roche, Basel, Switzerland). The medium was replaced with fresh complete DMEM at 16 h posttransfection, and the medium was harvested at 36 h posttransfection. The harvested lentiviral vector-containing medium was passed through a 0.22 μM filter and stored at −80 °C until use. Lentivirus infection was performed via the spinoculation method. The THP-1 and THP-1-ACE cells were seeded at 1.0 × 10^6^/mL in a 12-well plate in a complete RPMI medium supplemented with lentiviral vector solution (200 μL), TrunsDux and Max enhancer (System Biosciences, Palo Alto, CA, USA), after which the plate was centrifuged at 300 *×* *g* for 90 min at 32 °C. After spinoculation, the cells were washed with complete RPMI medium and seeded in 12-well plates. The positive control cells were selected with puromycin (1 μg/mL) beginning 48 h after viral inoculation. The cells were harvested on day 5 of selection and subjected to WB and real-time PCR to evaluate the efficiency of PPARα expression KD. The successfully generated PPARα-KD cells (more than 80% of the control cells) were used for subsequent experiments.

### Statistics

Student’s *t*-test and one-way analysis of variance (ANOVA) were used to analyze the data for significant differences. Values of *p* < 0.05, *p* < 0.01, and *p* < 0.001 were regarded as significant.

## Supplementary information

Supplementary materials

WB raw images
