# Supplementary material for: Peroxisome proliferator-activated receptor alpha is an essential factor in enhanced macrophage immune function induced by angiotensin-converting enzyme
Source: Cell Mol Immunol. 2025 Feb 5;22(3):243–59. doi: 10.1038/s41423-025-01257-y (PMC11868401; doi:10.1038/s41423-025-01257-y)
Supplement: Supplementary file 1 — Supplementary materials [file 41423_2025_1257_MOESM1_ESM.pdf]

Supplemental Materials

Peroxisome proliferator-activated receptor alpha is an essential factor in enhanced macrophage immune function induced by angiotensin converting enzyme

Suguru Saito, Duo-Yao Cao, Ellen A. Bernstein, Anthony E. Jones, Amy Rios, Aoi O. Hoshi, Aleksandr B. Stotland, Erika E. Nishi, Tomohiro Shibata, Jennifer E. Van Eyk, Ajit Divakaruni, Zakir Khan, Kenneth E. Bernstein.

Address correspondence to: Kenneth E. Bernstein, Professor, Department Pathology and Laboratory Medicine, Cedars-Sinai Medical Center, 8700 Beverly Blvd, Los Angeles, CA 90048, USA
Phone: +1-310-423-7562
E-mail: kbernst@cshs.org

Supplemental Methods

Immune activity assay in macrophages

To investigate the correlation between PPARα expression and immune activity of macrophages, thioglycolate-elicited peritoneal macrophages (TPMs) were isolated from WT, ACE 10/10 and ACE-KO mice, and used for *in vitro* experiments. The details of ACE 10/10 and ACE-KO were described in previous reports (5-12). CD80, CD86, H-2K^b (MHC class I) and I-A/I-E (MHC class II) expressions were measured in lipopolysaccharide (LPS)-stimulated TPMs (1.0x10^7/mL, at 37°C for 24 h) by flow cytometry. For measurement of antigen uptake, TPMs (1.0x10^7/mL) were incubated with FITC-labeled ovalbumin (OVA-FITC, 1 μg/mL) at 37°C for 2 h, then the uptake of OVA-FITC signal (mean fluorescence intensity; MFI) was analyzed by flow cytometry. For measurement of phagocytosis activity, TPMs (1.0x10^7/mL) were incubated with FITC-labeled Escherichia *coli* K-12 strain (*E. coli*-FITC, 25 μg/mL) at 37°C for 2 h, then the captured *E. coli*-FITC signal (MFI) was analyzed by flow cytometry. For antigen dependent T cells activation, *in vitro* antigen presentation assays were performed by using TPMs and splenic CD4+ or CD8+ T cells. Immunization was performed by subcutaneous (s.c) injection of 100 μl of OVA-Freund's Complete Adjuvant (FCA) emulsion (500 μg of OVA in 50% of FCA), then CD4+ or CD8+ T cells were isolated from the spleen of the mice at day 7 after immunization. The TPMs (5.0x10^5/mL) and CD4+ or CD8+ T cells (2.5x10^6/mL) (a ratio=1:5) were co-cultured in the presence of OVA (100 μg/mL) at 37°C for 24 h. The percentage of IFN-γ+CD4+ or CD8+ T cells was analyzed by flow cytometry. Cytokine production was assessed by in vitro stimulation and ELISA. TPMs (1.0x10^6/mL) were stimulated with LPS (1 μg/mL) at 37°C for 24 h, then IL-6, IL-12/IL-23p40 and TNF-α concentrations in the culture medium were measured by ELISA. Intranuclear PPARα expression levels were also measured by flow cytometry in each culture condition. The PPARα levels (fold MFI) and each value of immune activity were used to calculate correlation.

Metabolomic analysis

TPMs were washed with saline and the cell pellets were frozen at -80°C until subsequent treatment. For metabolite extraction, the cell pellets were treated with extraction buffer (80% methanol containing 0.85% ammonium bicarbonate). The samples were vortexed for 30 sec, then stored at -80°C for 30 min followed by centrifugation at 16,000 *g* for 10 min at 4°C, then supernatants were collected as metabolite containing fractions. The samples were treated by speedvac and dried samples were resuspended in 20% methanol. Metabolite extractions were analyzed with an Agilent 6470A Triple quadrupole mass spectrometer (Agilent Technologies, Santa Clara, CA, USA) operating in negative mode, connected to an Agilent 1290 Ultra High-Performance Liquid Chromatography (UHPLC) system (Agilent Technologies), and utilizing the MassHunter Metabolomics dMRM Database and Method to scan for 219 polar metabolites within each sample. The method is a highly reproducible and robust ion-pair reversed-phase (IP-RP) chromatographic method developed to provide separation of anionic and hydrophobic metabolites. Tributylamine (TBA), a volatile ternary amine amenable to electrospray ionization that functions as an ion pair reagent was used to facilitate and improve reproducible retention of acidic metabolites. The ion-pairing LC/MS method enables simultaneous analysis of multiple metabolite functional classes, including amino acids, citric acid cycle intermediates and other carboxylic acids, nucleobases, nucleosides, phosphorsugars, and fatty acids. Mobile phases consisted of HPLC or LCMS grade reagents. Buffer A is water with 3% methanol, 10 mM TBA, and 15 mM acetic acid. Buffers B and D are isopropanol and acetonitrile, respectively. Finally, Buffer C is methanol with 10 mM TBA and 15 mM acetic acid. The analytical column used was an Agilent ZORBAX RRHD Extend-C18 1.8 µm 2.1 x 150 mm coupled with a ZORBAX Extend Fast Guard column for UHPLC Extend-C18, 1.8 µm, 2.1 mm x 5mm. The MRM method takes advantage of known retention time information for each compound to create MRM transition lists that are dynamically created throughout an LC/MS run using a window around the expected retention times. In this way, compounds are only monitored while they are eluting from the LC, improving limits of detection, and permitting more metabolites to be measured within a short period of time. Resulting chromatograms were visualized in Agilent MassHunter Quantitative Analysis for QQQ. The final peaks were manually checked for consistent and proper integration. Results were analyzed in MetaboAnalyst 5.0 (http://www.metaboanalyst.ca).

Supplemental Tables

Supplemental Table 1. Primer sequences for genotyping PCR

Target	Primers
ACE	Forward: 5’-GACAGCTAA ACTTCC CGCG-3’<br>Reverse1: 5’-GTCCAGACTCATTCCAGAAC-3’<br>Reverse2: 5’-AGCAGCGACAGCATCAAGAG-3’
PPARα	Forward (Lf): 5’-AAAGCAGCCAGCTCTGTGTTGAGC-3’<br>Reverse (Er): 5’-TAGGTACCGTGGACTCAGAGCTAG-3’
LysM-Cre	Forward: 5’-GGACATGTTCAGGGATCGCCAGGCG-3’<br>Reverse: 5’-GCATAACCAGTGAAACAGCATTGCTG-3’

Supplemental Table 2. Antibody list for flow cytometry

Antigen	Fluorochrome	Clone	Reactivity	Company
CD45	PE, PB	30-F11	Mouse	BioLegend
CD11b	PE-Cy7, APC-Cy7	M1/70	Mouse	BioLegend
F4/80	BV650, PB, FITC	BM8	Mouse	BioLegend
Ly-6G	PB, FITC	1A8	Mouse	BioLegend
Ly-6C	APC	HK1.4	Mouse	BioLegend
CD11c	PB	N418	Mouse	BioLegend
CD3	Alexa488	17A2	Mouse	BioLegend
CD4	APC-Cy7	GK1.5	Mouse	BioLegend
D8	PE-Cy7, PerCP-Cy5.5	53-6.7	Mouse	BioLegend
B220	PE-Cy7	RA3-6B2	Mouse	BioLegend
CD80	FITC	16-10A1	Mouse	BioLegend
CD86	APC	GL-1	Mouse	BioLegend
I-A^b	FITC, PB	AF-120.1	Mouse	BioLegend
I-A/I-E	BV785	M5/114.15.2	Mouse	BioLegend
H-$2K^b$	FITC, PE, PB	AF6-88.5	Mouse	BioLegend
IFN-γ	PE	XMG1.2	Mouse	BioLegend
TNF-α	PerCP-Cy5.5, APC	MP6-XT22	Mouse	BioLegend
Granzyme B	PerCP-Cy5.5, PB	QA16A02	Mouse	BioLegend
IL-6	PE, FITC	MP5-20F3	Mouse	Thermo Fisher Scientific
IL-12/IL-23p40	PerCP-Cy5.5	C17.8	Mouse	Thermo Fisher Scientific
TRP-2/Tetramer	APC	-	Mouse	NIH Tetramer core

Supplemental Table 3. Primary antibody list for WB

Antigen	Catalog No.	Host	Dilution	Company
PPARα	MA1-822	Mouse	1:1000	Thermo Fisher Scientific
ACE	AF1513	Goat	1:1000	R&D Systems
β-actin	MA1-744	Mouse	1:10000	Thermo Fisher Scientific

Supplemental Table 4. Secondary antibody list for WB

Antigen	Catalog No.	Dilution	Company
IRDye® 680RD Goat anti-Mouse IgG Secondary Antibody	926-68070	1:12000	LI-COR Biosciences
IRDye® 800CW Goat anti-Mouse IgG Secondary Antibody	926-32210	1:12000	LI-COR Biosciences
IRDye® 680LT Donkey anti-Goat IgG Secondary Antibody	926-68024	1:12000	LI-COR Biosciences

Supplemental Table 5. Primer sequences for real-time qPCR

Target	Primers
Ppara	Forward: 5'-AGAGCCCCATCTGTCCTCTC-3'<br>Reverse1: 5'-ACTGGTAGTCTGCAAAACCAAA-3'
Cpt1a	Forward: 5'-CTCCGCCTGAGCCATGAAG-3'<br>Reverse: 5'-CACCAGTGATGATGCCATTCT-3'
Cpt1b	Forward: 5'-GCACACCAGGCAGTAGCTTT-3'<br>Reverse: 5'-CAGGAGTTGATTCCAGACAGGTA-3'
Cpt2	Forward: 5'-CAGCACAGCATCGTACCCA-3'<br>Reverse: 5'-TCCCAATGCCGTTCTCAAAAT-3'
Rxra	Forward: 5'-ATGGACACCAAACATTTCCTGC-3'<br>Reverse: 5'-CCAGTGGAGAGCCGATTCC-3'
Gapdh	Forward: 5'-TGTGTCCGTCGTGGATCTGA-3'<br>Reverse: 5'-TTGCTGTTGAAGTCGCAGGAG-3'

Supplemental Table 6. Identified pathways and Rich Ratios from IPA

For each pathway, Rich Ratios were calculated from RNA Seq TMP values (log2 (TPM+1) for the individual genes comprising the pathway. Genes considered in the analysis were significantly different ($p<0.05$) from WT levels as described in the Methods. FC is the fold change of A10-PPARα/WT divided by A10-PPARα-Cre/WT.

Lipid metabolism		Rich Ratio		
No.	Pathway	A10-PPARα/WT	A10-PPARα-Cre/WT	FC
#1	Synthesis of Lipoxins (LX)	0.2	0.167	1.197605
#2	Mitochondrial Fatty Acid Beta-Oxidation	0.189	0.054	3.49353
#3	Wax and plasmalogen biosynthesis	0.143	0.286	0.5
#4	Triglyceride metabolism	0.132	0.053	2.509506
#5	Ketone body metabolism	0.1	0	NA
#6	PI Metabolism	0.083	0.036	2.333333
#7	Vitamin D (calciferol) metabolism	0.083	0.083	1
#8	Sphingolipid metabolism	0.082	0.027	2.996337
#9	Carnitine metabolism	0.071	0.071	1
#10	Glycerophospholipid biosynthesis	0.07	0.039	1.797954
#11	Regulation of cholesterol biosynthesis by SREBP (SREBF)	0.067	0.067	1
#12	Regulation of lipid metabolism by PPARalpha	0.059	0	NA
#13	Fatty acyl-CoA biosynthesis	0.054	0	NA

#14	Metabolism of steroid hormones	0.053	0.026	2
#15	Arachidonic acid metabolism	0.044	0.022	2
#16	Peroxisomal lipid metabolism	0.035	0.035	1
#17	Bile acid and bile salt metabolism	0.022	0	NA

Immune system		Rich Ratio		
No.	Pathway	A10-PPARα/WT	A10-PPARα-Cre/WT	FC
#1	Rap1 signaling	0.25	0.188	1.329787
#2	Cytosolic sensors of pathogen-associated DNA	0.188	0.109	1.724771
#3	Signaling by CSF1 (M-CSF) in myeloid cells	0.161	0.065	2.496124
#4	MyD88 cascade initiated on plasma membrane	0.16	0.08	2
#5	Gene and protein expression by JAK-STAT signaling after IL-12 stimulation	0.135	0	NA
#6	FLT3 Signaling	0.128	0.077	1.664499
#7	Interleukin-17 signaling	0.125	0	NA
#8	STAT5 Activation	0.125	0.25	0.5
#9	Prolactin receptor signaling	0.118	0.118	1
#10	DAP12 interactions	0.116	0.023	4.978541
#11	Interleukin-1 family signaling	0.101	0.062	1.629032
#12	Interleukin-9 signaling	0.1	0.2	0.5
#13	Costimulation by the CD28 family	0.099	0.046	2.165939
#14	Alpha-protein kinase 1 signaling pathway	0.091	0.091	1
#15	Toll Like Receptor 3 (TLR3) Cascade	0.091	0.121	0.75124
#16	TNFR2 non-canonical NF-kB pathway	0.088	0.044	2
#17	Neutrophil degranulation	0.088	0.057	1.556537
#18	Interleukin-6 family signaling	0.083	0	NA
#19	NIK-->noncanonical NF-kB signaling	0.083	0.067	1.248876
#20	Butyrophilin (BTN) family interactions	0.083	0.083	1
#21	Other interleukin signaling	0.083	0.083	1
#22	Antimicrobial peptides	0.082	0.02	4
#23	Growth hormone receptor signaling	0.08	0.08	1
#24	MyD88-independent TLR4 cascade	0.079	0.105	0.751429
#25	Advanced glycosylation endproduct receptor signaling	0.077	0.077	1
#26	Signaling by the B Cell Receptor (BCR)	0.077	0.047	1.624204
#27	Class I MHC mediated antigen processing and presentation	0.076	0.068	1.115836
#28	C-type lectin receptors (CLRs)	0.076	0.076	1
#29	MyD88:MAL(TIRAP) cascade initiated on plasma membrane	0.071	0.119	0.6
#30	TNFs bind their physiological receptors	0.069	0.069	1
#31	Interleukin-1 processing	0.067	0.111	0.600901
#32	Interleukin-15 signaling	0.067	0.133	0.501504
#33	Signaling by CSF3 (G-CSF)	0.067	0.167	0.399401
#34	MyD88 dependent cascade initiated on endosome	0.065	0.129	0.5
#35	PKR-mediated signaling	0.064	0.09	0.714604

#36	TCR signaling	0.064	0.032	2.003155
#37	Interleukin-4 and Interleukin-13 signaling	0.063	0.045	1.402222
#38	Immunoregulatory interactions between a Lymphoid and a non-Lymphoid cell	0.056	0.014	3.985714
#39	MHC class II antigen presentation	0.055	0.055	1
#40	ISG15 antiviral mechanism	0.054	0.054	1
#41	MAP kinase activation	0.051	0.051	1
#42	Fcgamma receptor (FCGR) dependent phagocytosis	0.049	0.018	2.650273
#43	Regulation of TLR by endogenous ligand	0.046	0.046	1
#44	TAK1-dependent IKK and NF-kappa-B activation	0.046	0.114	0.399123
#45	Interleukin-7 signaling	0.042	0.083	0.5006
#46	Interferon gamma signaling	0.041	0	NA
#47	Interleukin-20 family signaling	0.039	0.077	0.50065
#48	DDX58/IFIH1-mediated induction of interferon-alpha/beta	0.037	0.086	0.428241
#49	Fc epsilon receptor (FCERI) signaling	0.037	0.034	1.076471
#50	Interleukin-3, Interleukin-5 and GM-CSF signaling	0.036	0.054	0.666045
#51	NLR signaling pathways	0.036	0.071	0.5
#52	MAPK targets/ Nuclear events mediated by MAP kinases	0.03	0.091	0.333333
#53	Defensins	0.022	0	NA
#54	Complement cascade	0.022	0.007	3.006803
#55	Interleukin-2 family signaling	0.021	0.043	0.5
#56	Interferon alpha/beta signaling	0.013	0.013	1
#57	OAS antiviral response	0	0.111	0
#58	SLC15A4:TASL-dependent IRF5 activation	0	0.167	0

Supplemental Figures

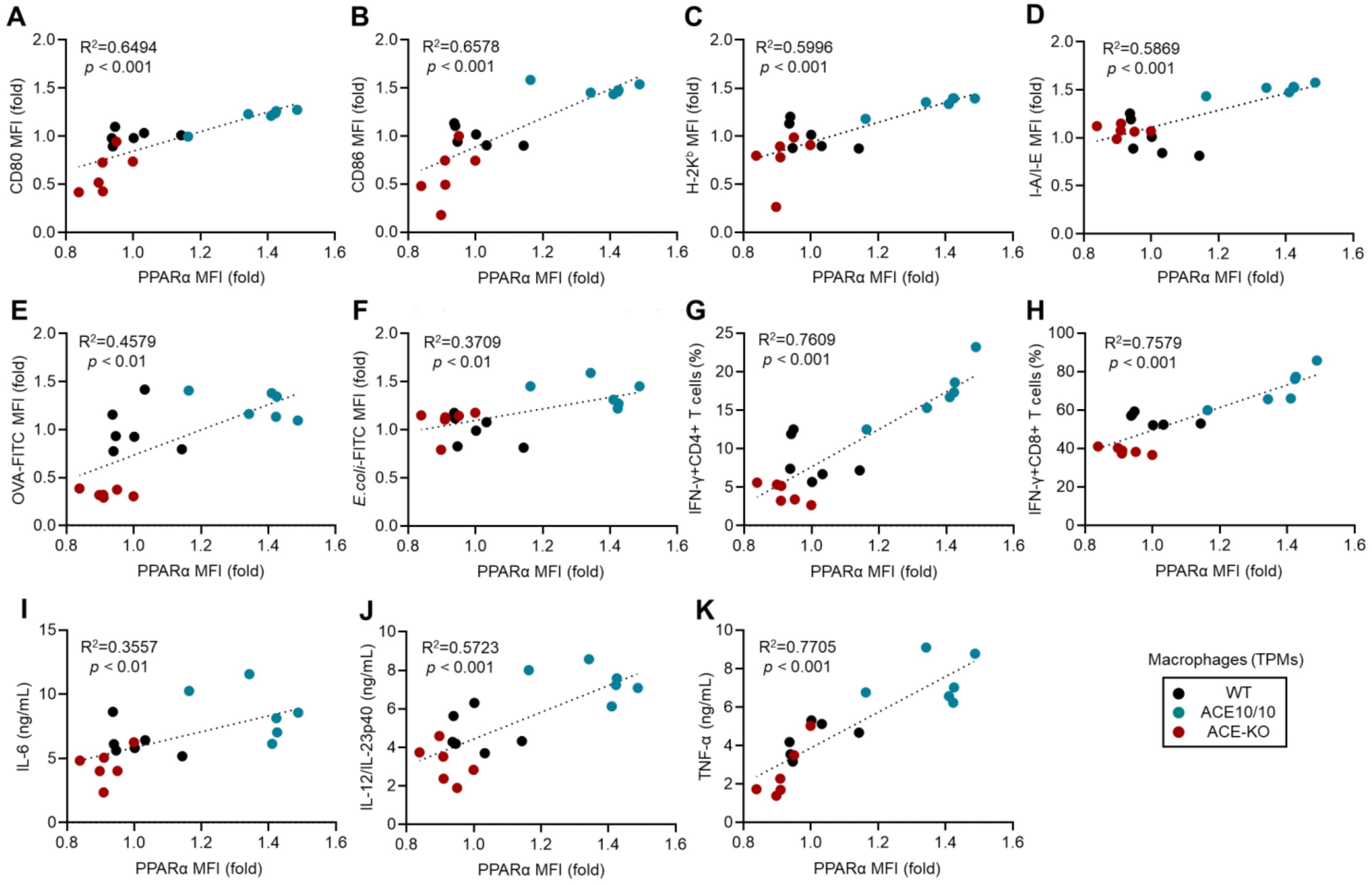

Supplemental Figure 1. Correlation of PPARα expression level and immune function in macrophages
The PPARα expression level and immune activity were measured in TPMs by following protocols described in supplemental methods. The values were used for generation of linear regression and calculation of correlations. The correlations were represented as between PPARα MFI and (A) CD80 MFI, (B) CD86 MFI, (C) H-2K^b, (D) I-A/I-E MFI, (E) incorporated OVA-FITC MFI, (F) captured *E. coli*-FITC MFI, (G) IFN-γ$^+$CD4$^+$ T cells (%), (H) IFN-γ$^+$CD8$^+$ T cells (%), (I) IL-6 production (ng/mL), (J) IL-12/IL-23p40 production (ng/mL) or (K) TNF-α production (ng/mL). All MFI values are represented as fold changes (the average value of WT was used for base=1). The linear regressions were generated and R-squared and p-value were calculated by using GraphPad Prism 10.0. The values $p < 0.05$, $p < 0.01$ and $p < 0.01$ were considered as significant difference.

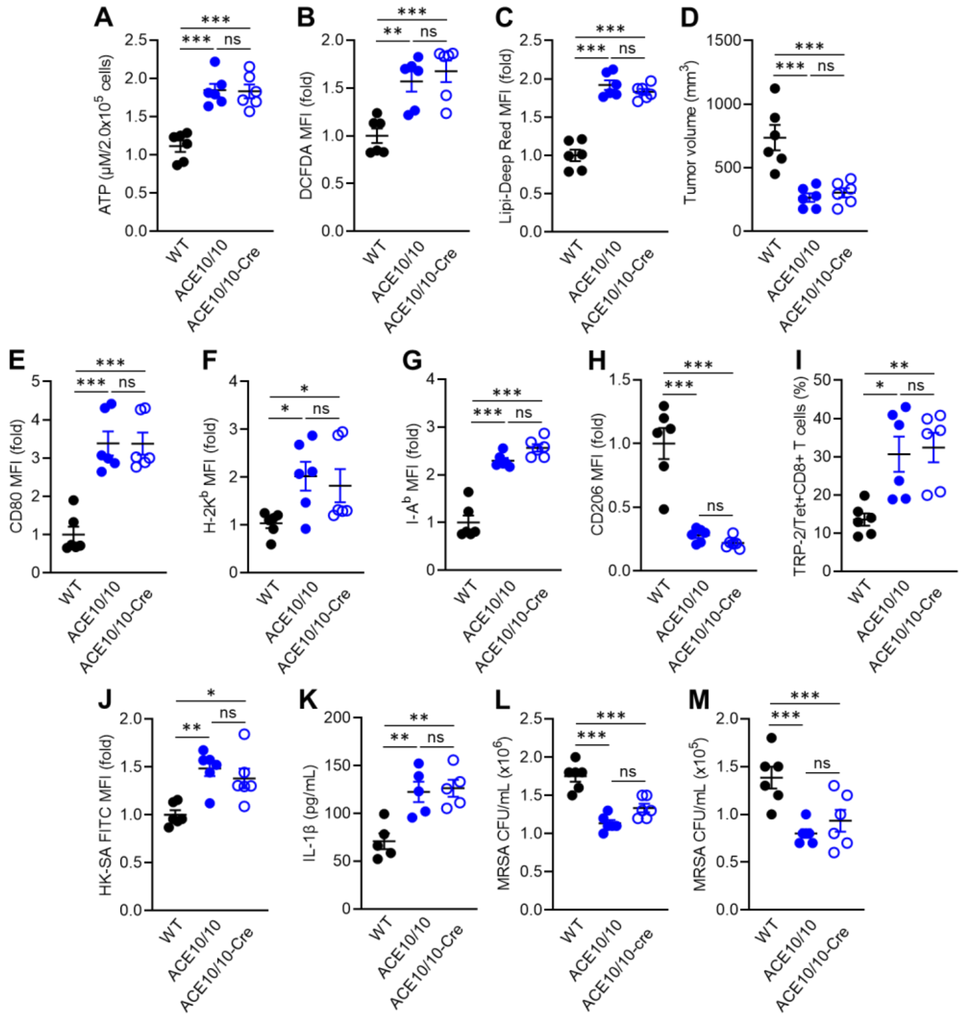

Supplemental Figure 2. Similar metabolism and immune response in ACE 10/10-Cre mice to original ACE 10/10 mice

A) ATP level in macrophages. TPMs (naive) were treated with CellTiter Glo® 2.0 and intracellular ATP concentration was measured by luminescence. The graph shows ATP concentrations (μM) per 2.0x10^5 cells. B) ROS production in macrophages. TPMs (naive) were stained with DCFDA for ROS detection and analyzed by flow cytometry. The graph shows MFI values (fold change) of DCFDA. C) Lipid uptake assay in macrophages. TPMs (naive) were treated with OA (200 μM) at 37°C for 16 h followed by Lipi-Deep Red (LDR) staining for flow cytometry. The graph shows MFI values (fold change) of LDR. D) B16-F10 tumor challenge. The mice received s.c. injection of B16-F10 cells (100 μl of 1.0x10^7/mL in PBS) and tumor volumes were measured on day 14. E-I) Characterization of intratumor (IT) immune cells on day 14 of tumor bearing mice. The graphs show MFI values (fold change) of (E) CD80, (F) H-2K^b, (G) I-A^b, (H) CD206 and (I) percentages of TRP-2/Tet$^+$CD8$^+$ T cells. J) *In vitro* phagocytosis assay. TPMs were incubated with HK-SA-FITC (1.0x10^8 CFU/mL) at 37°C for 2 h. The bacterial phagocytosis was analyzed by flow cytometry. The graph shows MFI values (fold change) of HK-SA-FITC in the TPMs. K) *In vitro* stimulation and cytokine production assay. TPMs were stimulated with HK-SA (1.0x10^7 CFU/mL) at 37°C for 24 h. The IL-1β concentration in the culture medium was measured by ELISA. L-M) *In vitro* MRSA killing. TPMs were incubated with MRSA (1.0x10^8 CFU/mL) at 37°C for 5 h. The MRSA survival were quantified by measuring CFUs in supernatant (L) and intracellular (M). The cumulative data are shown as mean ± SEM values of six samples from two independent experiments. All MFI values are represented as fold changes (the average value of WT was used for base=1). One-way ANOVA was used to analyze data for significant differences, and *$p < 0.05$, **$p < 0.01$ and ***$p < 0.01$ were considered as significant difference. ns is not significant.

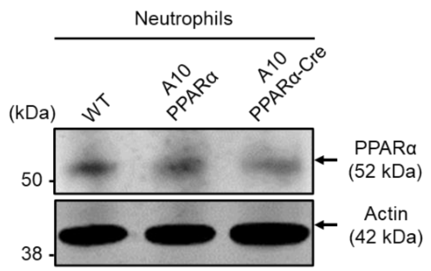

Supplemental Figure 3. PPARα expression in neutrophils

Representative Western blot of PPARα in bone marrow (BM)-isolated neutrophils originating from WT, A10-PPARα and A10-PPARα-Cre mice.

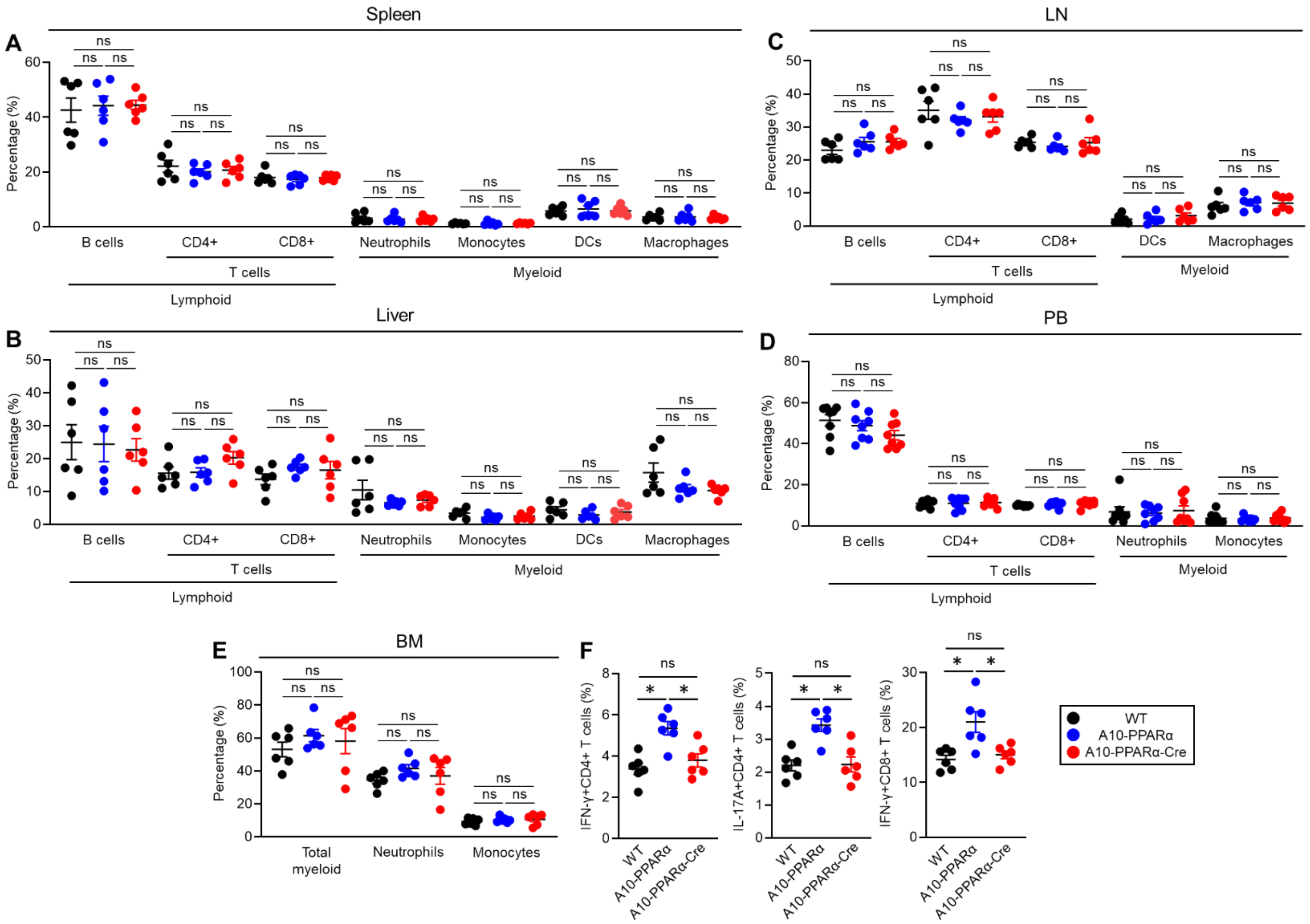

Supplemental Figure 4. Screening of immune cells

The percentage of each immune cell population was analyzed by flow cytometry in represented organs and tissues, such as A) spleen, B) liver, C) lymph node (LN), D) peripheral blood (PB), E) bone marrow (BM). The effector function of splenic T cells was assessed by cytokine productions. The percentages of IFN-γ^+CD4$^+$ T cells (Th1), IL-17A$^+$CD4$^+$ T cells (Th17) and TNF-α^+CD8$^+$ T cells (Tc) were shown as (F). The cumulative data are shown as mean ± SEM values of six samples from two independent experiments. One-way ANOVA was used to analyze data for significant differences, and *p < 0.01 was considered as significant difference. ns is not significant.

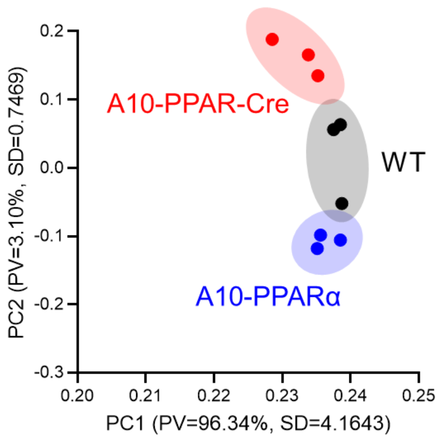

Supplemental Figure 5. PCA plot of TPM gene clusters

PCA plot was generated by using RNA sequencing data of TPMs in each group of mice.

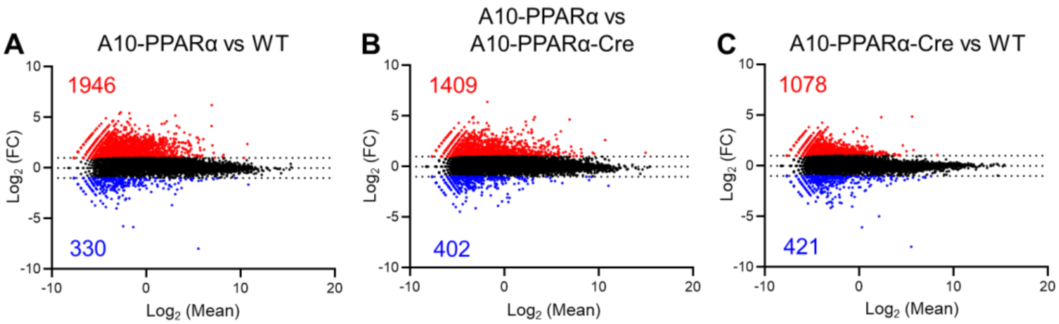

Supplemental Figure 6. Gene expression differences in macrophages identified by RNA sequencing

MA plots were generated by following $\log_2$ (Mean) and $\log_2$ (Fold Change; FC) of transcripts per million (TPM) values in the represented comparisons, A) A10-PPARα vs WT, B) A10-PPARα vs A10-PPARα-Cre and C) A10-PPARα-Cre vs WT. The number of upregulated (FC ≥ 2, red) and downregulated (FC ≤ 0.5, blue) genes are indicated on the plots.

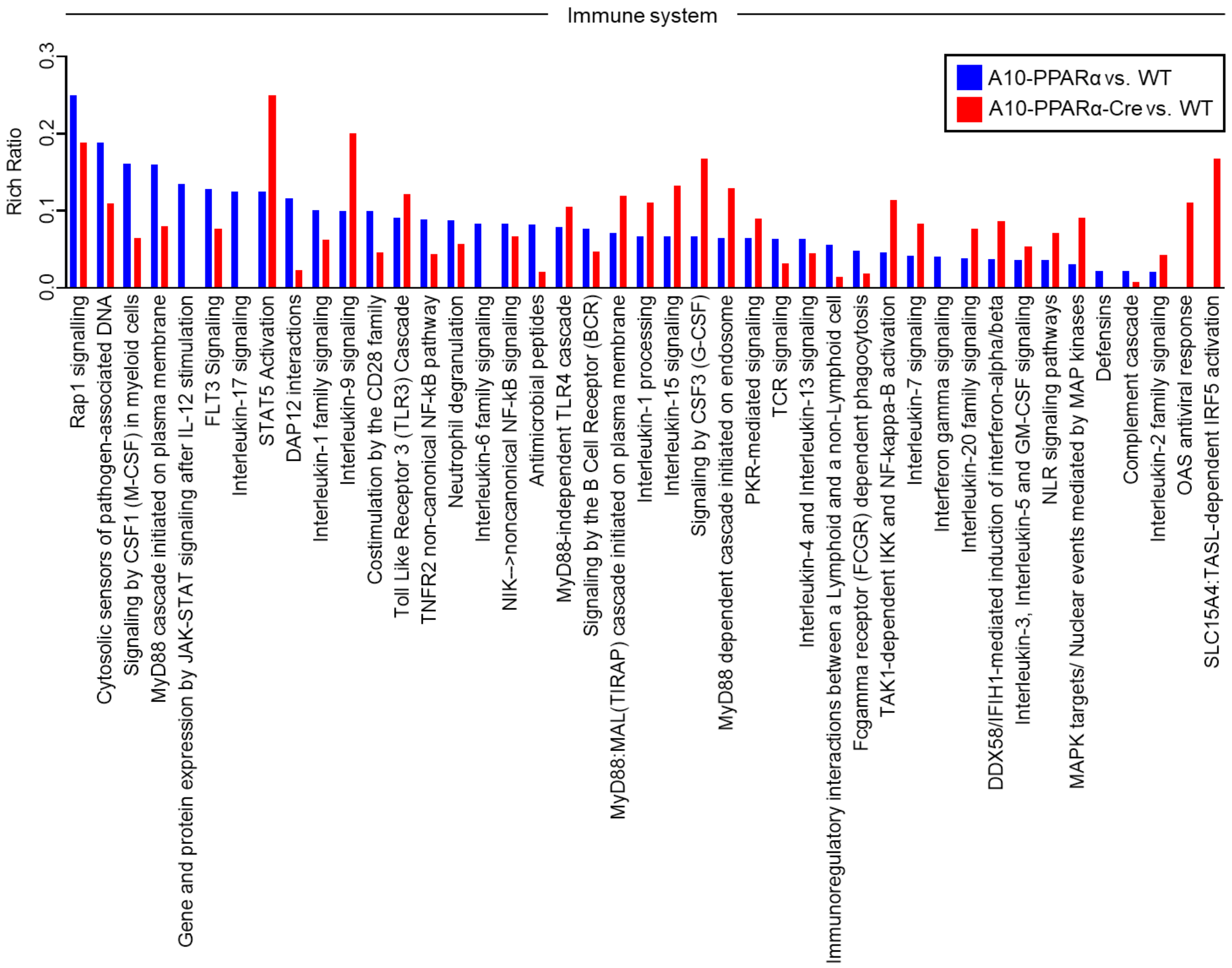

Supplemental Figure 7. PPARα depletion alters the gene expression associated with immune system pathways in macrophages

Similar to Fig. 2A, total RNA was isolated from TPMs of WT, A10-PPARα, or A10-PPARα-Cre mice (n=3 in each group) and subjected to bulk RNA sequencing. The TPM values obtained from RNA sequencing were used for analysis after conversion to log2 (TPM+1) values. Immune system pathways were identified using IPA, based on genes with significant expression changes (p<0.05) in either of two independent comparisons: A10-PPARα-Cre vs. WT or A10-PPARα-Cre vs. WT. The Rich Ratio for each pathway was also calculated by IPA. The figures presented show the identified pathways in which the Rich Ratio for A10-PPARα-Cre vs. WT is either greater than 1.2-fold or less than 0.8-fold compared to A10-PPARα-Cre vs. WT. Additionally, pathways with a Rich Ratio of 0 for A10-PPARα-Cre vs. WT are also shown.

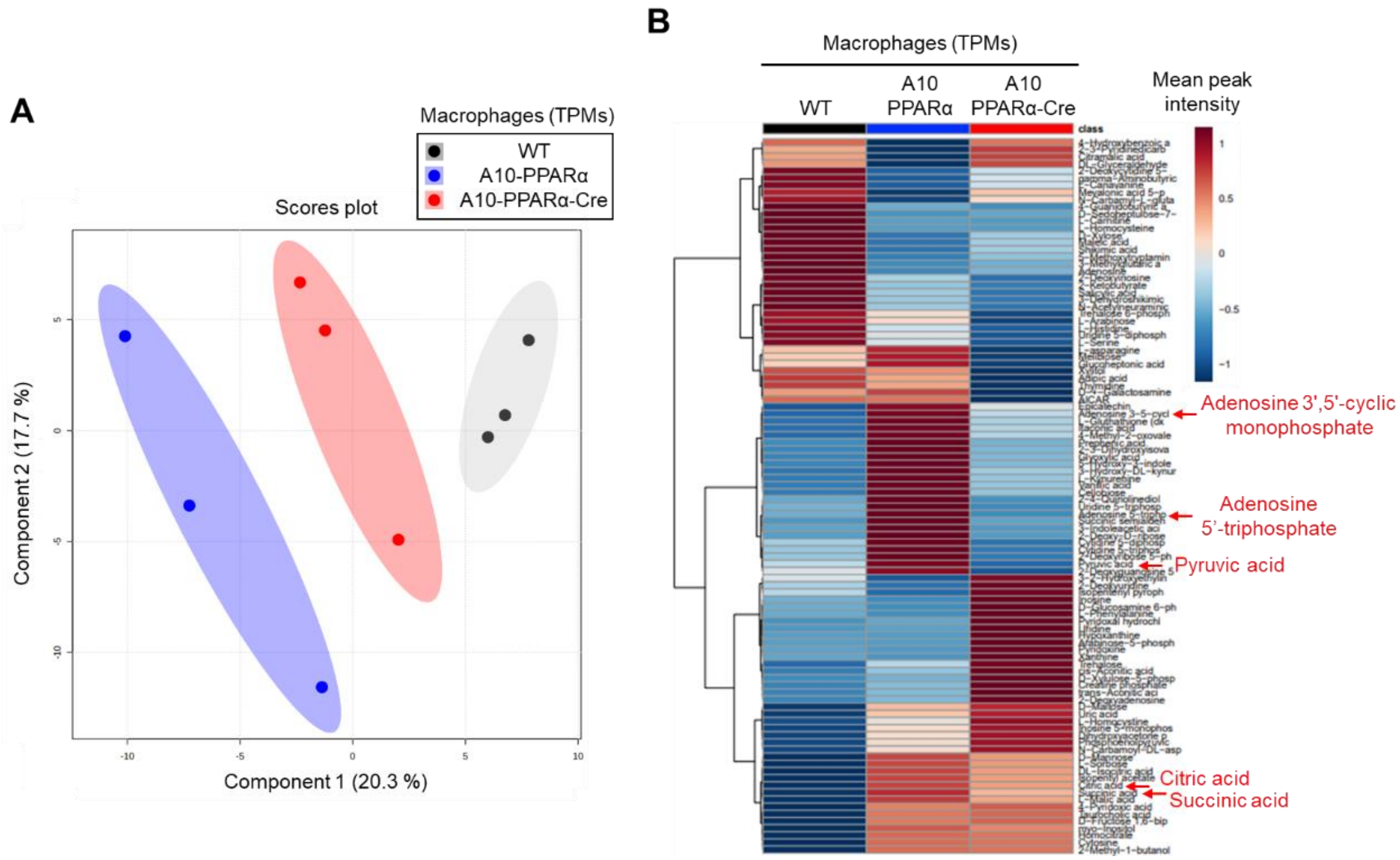

Supplemental Figure 8. Metabolomic analysis in macrophages

TPMs were collected from WT, A10-PPARα and A10-PPARα-Cre mice and subjected to metabolomics. A) Partial least squares-discriminant analysis (PLS-DA) plots for metabolome comparison between the three different groups of macrophages. Single plot shows the data obtained from TPMs originating from a single mouse. B) Heatmap of the top 100 identified metabolites in three groups of macrophages. The heatmap was generated by following the mean peak intensity values of each group of macrophages. The metabolomic data was analyzed and PLS-DA plot and heatmaps were generated by MetaboAnalyst 5.0.

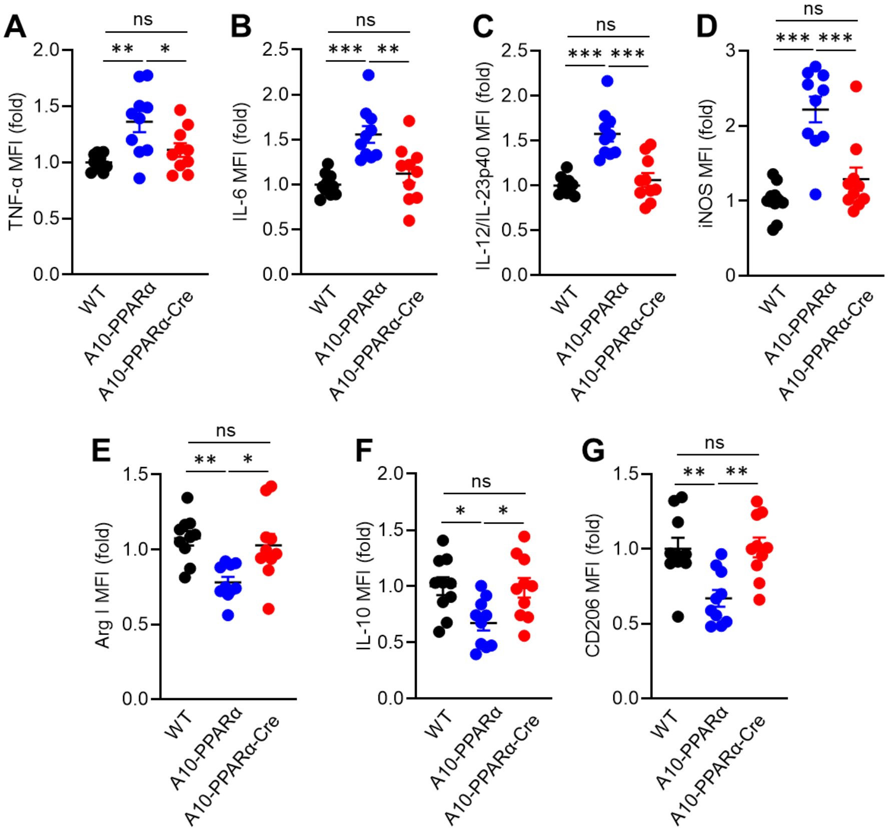

Supplemental Figure 9. The functional analysis of IT macrophages

The tumor-derived cells were isolated from B16-F10 originated tumor baring mice in day 14 of post tumor inoculation. The samples were subjected to macrophage functional analysis in flow cytometry. The MFI values (fold change) of TNF-α (A), IL-6 9B), IL-12/Il-23p40 (C), iNOS (D), Arg I (E), IL-10 (F) and CD206 (G) were presented. The cumulative data are shown as mean ± SEM values of ten samples from three independent experiments. All MFI values are represented as fold changes (the average value of WT was used for base=1). One-way ANOVA was used to analyze data for significant differences, and **p* < 0.05, ***p* < 0.01 and ****p* < 0.01 were considered as significant difference. ns is not significant.

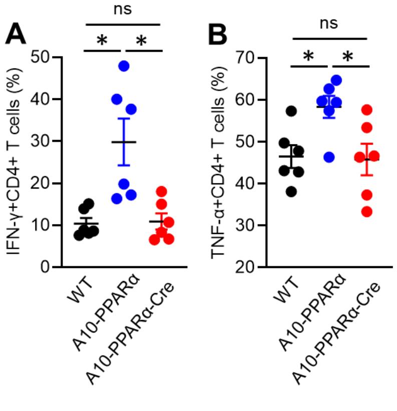

Supplemental Figure 10. Cytokine productions of IT $CD4^+$ T cells

The tumor-derived cells were isolated from B16-F10 originated tumor baring mice in day 14 of post tumor inoculation. The samples were stimulated with PMA (100 ng/mL) and ionomycin (250 ng/mL) in the presence of Golgi Stop^{TM} (1 μg/mL) at 37 °C for 5 h prior to analyze the samples by flow cytometry. The percentage of IFN-γ (A) and TNF-α (B) producing $CD4^+$ T cells were presented. The cumulative data are shown as mean ± SEM values of six samples from two independent experiments. One-way ANOVA was used to analyze data for significant differences, and **p* < 0.05 was considered as significant difference. ns is not significant.

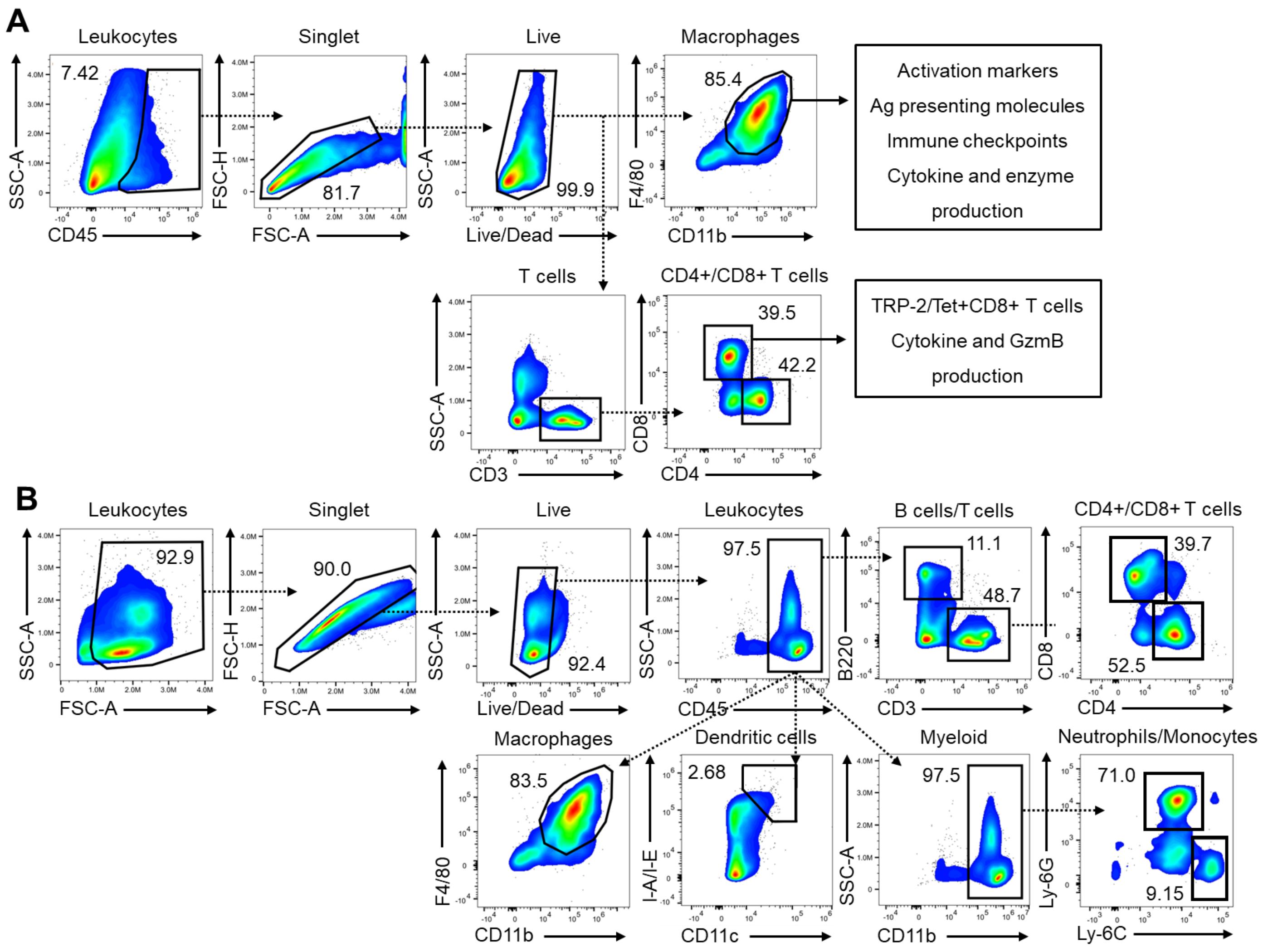

Supplemental Figure 11. Gating strategy of flow cytometry analysis

Flow cytometry analyses were performed by staining with fluorochrome-conjugated mAb or tetramer following the gating strategies shown above. A) Gating strategy for analysis of intratumor (IT) macrophages and CD8$^+$ T cells. Total leukocyte population was first gated on CD45$^+$, then live cells were separated as macrophages (CD45$^+$CD11b$^+$F4/80$^+$) or CD8$^+$ T cells (CD45$^+$CD3$^+$CD8$^+$). The cell surface markers, cytokines, iNOS, and arginase I (Arg I) expressions were measured in the macrophage gate. The TRP-2/Tet$^+$ population was determined in the CD8$^+$ T cell gate. Further, cytokine and granzyme B (GzmB) productions were measured in TRP-2/Tet$^+$CD8$^+$ T cells. B) Gating strategy for characterization of immune cells in spleen, liver, lymph node (LN), bone marrow (BM) and peripheral blood (PB). Total leukocytes were first gated on the FSC-A/SSC-A panel followed by determination of CD45$^+$ cells in live cells. The population was subsequently separated to CD11b$^+$ (myeloid), CD11b$^+$F4/80$^+$ (macrophages), CD11c$^+$MHC class II^{hi} (dendritic cells; DCs), CD3$^+$ (T cells) or CD19$^+$ (B cells). The CD11b$^+$ population was further classified as neutrophils (Ly-6G+Ly-6C$^{mid/+}$) or monocytes (Ly-6G$^-$ Ly-6C^{hi}). The CD3$^+$ population was further classified to CD4$^+$ or CD8$^+$ T cells.
