# Supplementary figures and images for: Peroxisome proliferator-activated receptor alpha is an essential factor in enhanced macrophage immune function induced by angiotensin-converting enzyme
Source: Cell Mol Immunol. 2025 Feb 5;22(3):243–59. doi: 10.1038/s41423-025-01257-y (PMC11868401; doi:10.1038/s41423-025-01257-y)

Source WB image

Figure 1C

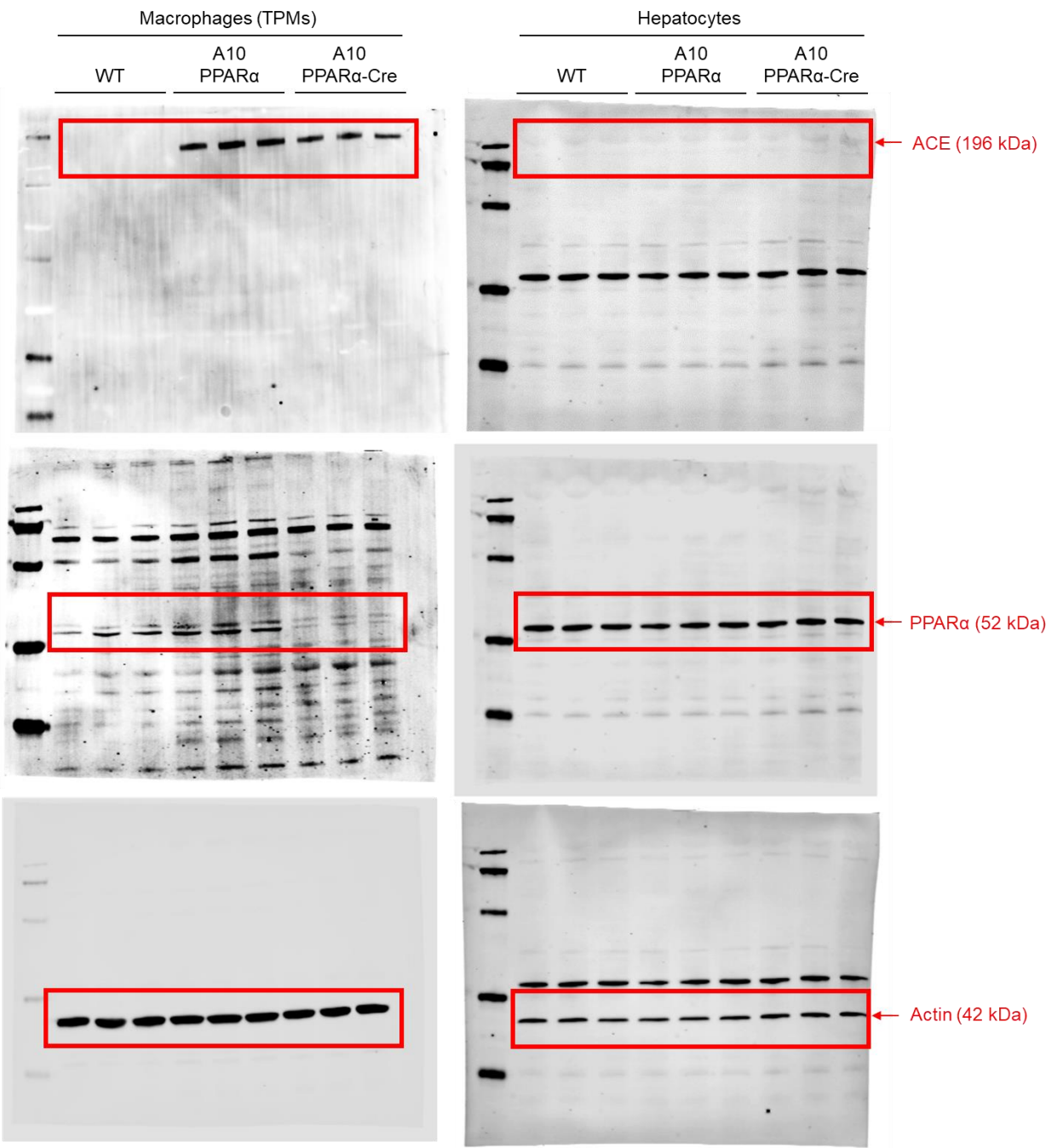

Supplemental Figure 2

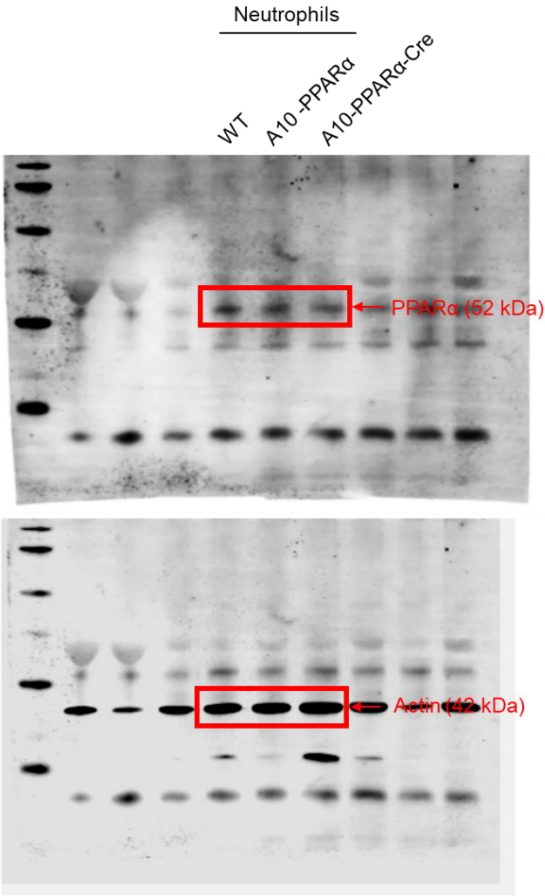

Supplement: Supplementary file 2 — WB raw images [file 41423_2025_1257_MOESM2_ESM.pdf]
